# Synthesis, Crystal Structure, Hirshfeld Surface Analysis, and Computational Approach of a New Pyrazolo[3,4-*g*]isoquinoline Derivative as Potent against Leucine-Rich Repeat Kinase 2 (LRRK2)

**DOI:** 10.1021/acsomega.4c03208

**Published:** 2024-07-01

**Authors:** Etify A. Bakhite, Shaaban Kamel Mohamed, Chin-Hung Lai, Karthikeyan Subramani, Islam S. Marae, Suzan Abuelhassan, Abdelhamid A. E. Soliman, Mohamed S. K. Youssef, Hatem A. Abuelizz, Joel T. Mague, Rashad Al-Salahi, Youness El Bakri

**Affiliations:** † Department of Chemistry, Faculty of Science, Assiut University, Assiut 71516, Egypt; ‡ Chemistry and Environmental Division, Manchester Metropolitan University, Manchester M1 5GD, England; § Chemistry Department, Faculty of Science, Minia University, El-Minia 61519, Egypt; ∥ Department of Medical Applied Chemistry, Chung Shan Medical University, Taichung 40241, Taiwan; ⊥ Department of Medical Education, Chung Shan Medical University Hospital, Taichung 40201, Taiwan; # Center for Healthcare Advancement, Innovation and Research, Vellore Institute of Technology University, Chennai Campus, Chennai 600127, India; ∇ Department of Chemistry, Tulane University, New Orleans, Louisiana 70118, United States; ○ Department of Pharmaceutical Chemistry, College of Pharmacy, King Saud University, Riyadh 11451, Saudi Arabia; ◆ Department of Theoretical and Applied Chemistry, South Ural State University, Lenin prospect 76, Chelyabinsk 454080, Russian Federation

## Abstract

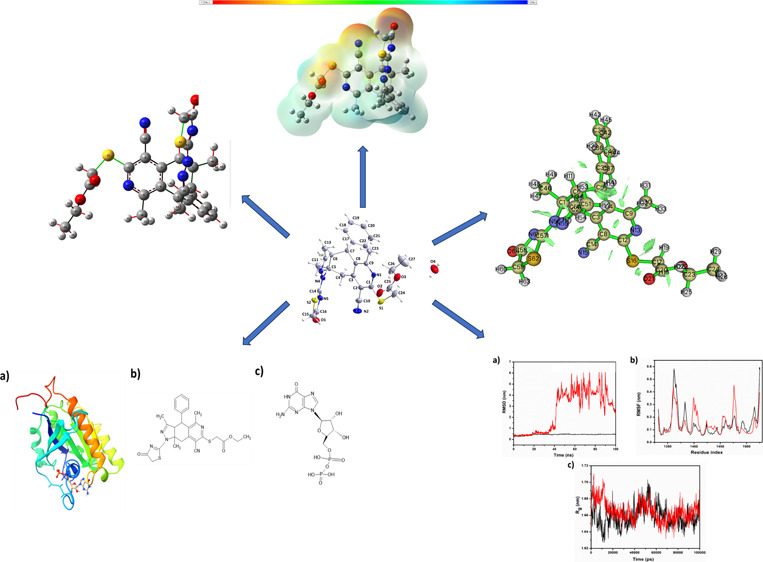

Ethyl-2-((8-cyano-3,5,9a-trimethyl-1-(4-oxo-4,5-dihydrothiazol-2-yl)-4-phenyl-3a,4,9,9a-tetrahydro-1*H*-pyrazolo[3,4-*g*]isoquinolin-7-yl)thio)acetate (**5**) was synthesized, and its structure was characterized by IR, MS, and NMR (^1^H and ^13^C) and verified by a single-crystal X-ray structure determination. Compound **5** adopts a “pincer” conformation. In the crystal, the hydrogen bonds of −H···O, C–H···O, and O–H···S form thick layers of molecules that are parallel to (101). The layers are linked by C–H···π(ring) interactions. The Hirshfeld surface analysis shows that intermolecular hydrogen bonding plays a more important role than both intramolecular hydrogen bonding and π···π stacking in the crystal. The intramolecular noncovalent interactions in **5** were studied by QTAIM, NCI, and DFT-NBO calculations. Based on structural activity relationship studies, leucine-rich repeat kinase 2 (LRRK2) was found to bind **5** and was further subjected to molecular docking studies, molecular dynamics, and ADMET analysis to probe potential drug candidacy.

## 1 Introduction

The goal of current Parkinson’s disease (PD) therapeutic approaches is to reestablish dopamine signaling to improve motor functions.^1^ These approaches are based on groundbreaking research by Arvid Carlsson, who showed how, in animal studies, injecting l-dihydroxyphenylalanine (l-DOPA), a precursor to dopamine biosynthesis, could restore motor function.^2^ Until recently, the cornerstone of PD treatment has been comparable to dopamine receptor agonists along with oral l-DOPA. However, it took nearly a decade to establish a good dose and administration schedule of these for PD in human patients.^3^

Novel therapy approaches are required for PD. Furthermore, this requirement is increasing because of the aging of the global population.^4^ Thus, much research has focused on the genetic origins of Parkinson’s disease (PD) in the hopes that they could lead to new targets for medications in the future. Treatment plans that focus on proteins implicated in the genesis of Parkinson’s disease have considerable promise. These strategies might, in theory, both stop the advancement of Parkinson’s disease and even be enough to stop it from happening in the first place.

The LRRK2 gene produces the leucine-rich repeat kinase 2 protein (LRRK2). An area on chromosome 12 linked to autosomal dominant late-onset parkinsonism is known as the PARK8 locus, where LRRK2 was first identified separately by two groups.^5,6^ As per Kluss et al.^7^ and Nalls et al.,^8^ LRRK2 mutation has been recognized to be among the most common contributors to familial PD. Thus, promoting LRRK2 kinase activity in PD *in vivo* and enhanced LRRK2 interaction with GTP and PD are correlated. Although the exact relationship between these pathways is still unknown, biochemical data suggest that the two enzymatic functions of LRRK2 are somewhat reliant on one another.

The identification of inhibitors that compete with ATP for LRRK2 and demonstrate selectivity for the pathogenic G2019S LRRK2 variation compared to the wild-type LRRK2 represents a noteworthy recent development.^9^ Using a combination of systematic chemical changes, *in silico* docking investigations, as well as compound library screening, Garofalo et al.^9^ found many indazole compounds that exhibit selectivity for G2019S. Greater than 300 times more efficacy in cellular tests and greater than 200 times better potency *in vitro* were found for the most selective molecule. This is remarkable “given that G2019S- It is clear that a large number of interesting ATP-competitive LRRK2 kinase inhibitors are being developed. Most of these compounds will undoubtedly fail as PD therapies, but there are reasons to be hopeful because a wide range of chemical structures are accessible. A wider range of pharmacological characteristics and an increased likelihood of discovering a medication that is effective, selective, and devoid of side effects are associated with diverse chemical structures. Differing from WT-LRRK2 only by a single amino acid,” as the authors themselves correctly note.^9^ In addition to this, the 5,6,7,8-tetrahydroisoquinoline ring system is a structural fragment of many alkaloids that are next to indole alkaloids in their abundance.^10,11^ Compounds containing a 5,6,7,8-tetrahydroisoquinoline fragment are used as intermediate products in the synthesis of alkaloids,^12^ precursors to enzyme inhibitors,^13^ fungicides,^14^ potassium receptor antagonists,^15^ and drugs for the treatment of cardiovascular diseases, bronchial asthma, tumors, and viral infections.^16^ 5,6,7,8-Tetrahydroisoquinoline derivatives were also shown to exhibit anticonvulsants,^17^ antibacterial,^18^ neurotropic,^19^ and antimicrobial activities.^20^ Herein we report that **5** exhibits potent inhibition against LRRK2 based on structural activity relationship studies and give the results of various computational and structural analyses to explore potential drug candidacy.

## 2 Experimental Details

### 2.1 Instrumentation

The melting point of **5** was determined on a GallanKamp apparatus and is uncorrected. Its FTIR spectrum was recorded on a Shimadzu 470 IR-spectrophotometer (KBr; ν_max_ in cm^–1^). The NMR spectra were recorded on a Bruker (400 MHz for ^1^H NMR and 100 MHz for ^13^C NMR) spectrometer using CDCl_3_ as a solvent and tetramethylsilane (TMS) as an internal reference.

### 2.2 Single-Crystal X-ray Analysis

A suitable crystal of **5** was mounted on a polymer loop with a drop of heavy oil and placed in a cold nitrogen stream on a Bruker D8 QUEST PHOTON 3 diffractometer. Intensity data were collected under the control of the *APEX3*(21) software, and the raw data were reduced to *F*^2^ values with *SAINT*,^22^ which also performed a global refinement of unit cell parameters. Application of a numerical (face-indexed) absorption correction and merging of equivalent reflections were carried out with *SADABS,*(23) and the structure was solved by dual space methods (*SHELXT*^24^). The structural model was refined by full-matrix, least-squares procedures (*SHELXL*^25^) with hydrogen atoms attached to carbon included as riding contributions with isotropic displacement parameters tied to those of the attached atoms. Those of the disordered lattice water molecules were placed to maximize their hydrogen bonding abilities and included also as riding contributions. The ethyl group was disordered over two sites, and the components were refined subject to restraints that their geometries be comparable. Crystal and refinement data are presented in Table S1.

### 2.3 Hirshfeld Surface (HS) Analysis

The HS analysis is a means of defining and quantifying intermolecular interactions in crystalline compounds. Spackman et al. proposed a method to divide the distribution of electrons within a molecule in the crystalline phase into several parts based on Hirshfeld’s partitioning scheme.^26−28^ The CrystalExplorer software (version 17)^29^ was used to generate and analyze the HS of **5** using the experimental (X-ray) file as input.

### 2.4 DFT and NBO Studies

The DFT study examined the gas-phase structure of **5** employing the hybrid B3LYP functional that is a combination of the exact exchange (HF) and Becke functional as well as the LYP correlation functional based on Becke’s idea^30−32^ in conjunction with the 6-311++G** basis set.^33^ After the converged geometry was obtained, the vibrational harmonic frequencies were calculated at the same theoretical level to ensure that the imaginary frequency number was zero for the stationary point. For the study of the intrinsic electronic properties of **5**, the NBO analysis was performed at the same theoretical level. All calculations were performed with Gaussian 16.^34^

### 2.5 The QTAIM Study

The quantum theory of atoms in molecules (QTAIM), also called atoms in molecules (AIM), is a model of molecules and condensed matter. In this model, the major objects of molecules and condensed matter, i.e., atoms and bonds, are expressed by the distribution function of the observable electronic density of a molecule, which is a probability distribution and describes the average distribution of the electronic charge in the field of attractions exerted by the nuclei. According to QTAIM, the molecular structure is revealed by the stationary points and gradient paths of electron density. The gradient paths of the electron density of a molecule originate and terminate from the stationary points. In this study, the QTAIM analysis was performed using the Multiwfn program.^35^

### 2.6 The Noncovalent Interaction Index (NCI) Study

To visualize noncovalent interactions in a molecule/complex, such as van der Waals interactions, hydrogen bonds, and steric clashes, NCI is a useful tool based on the electron density and its derivatives.^36^

Using the density and its first derivative, the reduced density gradient (*s*(*r*)) can be calculated as
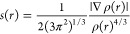
1

The dimensionless function *s*(*r*) could be used to describe the deviation from a homogeneous electron distribution. In remote regions of the molecule, where the density decays exponentially to zero, the reduced gradient has very large positive values. In the regions in which covalent bonding or noncovalent interactions form, the reduced gradient has a value close to zero. In this study, the NCI index based on *s*(*r*) was obtained by the Multiwfn program.^35^

### 2.7 Reactivity Descriptors Derived from the Conceptual Density Functional Theory

Local reactivity descriptors (LRDs), for example, the local electrophilicity index, originated from conceptual density functional theory (CDFT) and arose from a perturbational approach. These were the initial responses of a particular fragment to the perturbation caused by the approach of a second moiety. LRDs have already been used as convenient and powerful tools to investigate the reactivity of some intra- or intermolecular reactions.^31,37−46^ Upon considering a finite difference approximation, the electronic chemical potential (μ) correlating with the escaping tendency of an electron from a system can be expressed by the following equation:
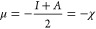
2where χ, *I*, and *A* represent the electronegativity, ionization potential, and electron affinity, respectively. Within the premise of the hard–soft acid–base principle, the chemical hardness (η) can be expressed as follows:
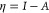
3

Based on CDFT, the electrophilicity (ω) of a molecule can be expressed as follows:
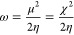
4

According to the CDFT, μ, η, and ω belong to global reactivity descriptors (GRDs). To understand the reactivity of a specific atom within a molecule, GRDs and several LRDs have already been developed. For example, the Fukui function was developed and defined as follows:
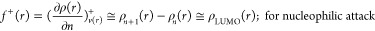
5
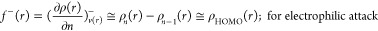
6where ρ(*r*), *n*, and *v*(*r*) are the electron density, the number of electrons, and the external potential, respectively. The Fukui functions of **5** were obtained using the Multiwfn program.^35^

### 2.8 The Average Local Ionization Energy (ALIE) Analysis

The average local ionization energy can be calculated by eq 7:
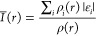
7where ρ_*i*_(*r*) and ε_*i*_ are the electron density function and orbital energy of the *i*th molecular orbital, respectively. The average local ionization energy is a widely used parameter, for example, revealing atomic shell structures, measuring electronegativity, predicting p*K*_a_, and quantifying local polarizability and hardness.^47,48^ Its most important application is to determine which parts of a molecule are susceptible to attack by electrophiles or free radicals. In a molecule, if a site has a smaller value of the average local ionization energy, it means that electrons are less bound at this site and that this site is more vulnerable to electrophilic or free radical attacks. In this study, the average local ionization energy analysis used the Multiwfn program.^35^

### 2.9 Molecular Docking Studies

Molecular docking techniques were used to predict and assess the binding interactions between the structure of the leucine-rich repeat kinase 2’s ROC domain interacting with the microtubule facing the plus end (LRRK2) using the GDP unique ligand. The crystal structure of LRRK2 was obtained from the Research Collaboratory for Structural Bioinformatics (RCSB’s) protein data bank^49,50^ through the protein ID (https://www.rcsb.org/structure/7THZ).^51^ The LRRK2 protein is associated with a native ligand and guanosine-5′-diphosphate. The structures of both the compound and its native ligand were drawn using ChemSketch,^52^ and LRRK2 was preprocessed using the Protein Prep Wizard panel to remove unwanted heteroatoms and lattice water molecules. Following this, optimization and minimization procedures were applied to the structure with the OPLS_2005 (Optimized Potentials for Liquid Simulations) force field.^53^ Similarly, **5** was minimized through the OPLS_2005 force field by the Ligprep application. With the preprocessed input structure, molecular docking simulations were carried out using Autodock4.^54^ To prepare the LRRK2-**5** complex for simulations, hydrogen atoms, Kollman charges, and Gasteiger charges were added to the respective molecules. The docking procedure maintained the rigidity of the LRRK2 protein while treating the ligands as completely flexible. During blind docking simulations, 100 distinct complex conformations were assigned to the grid box surrounding the entire protein. Upon completion of the docking simulations, a series of dockings were generated and stored in the DLG file. The best pose was determined based on the most negative binding energy. The CHARMM-GUI website was used to generate input for molecular dynamics using the best pose.

### 2.10 Molecular Dynamics Studies

The CHARMM 36 force field through the online server (https://www.charmm-gui.org/)^55,56^ was used to create a topology for the LRRK2-**5** complex, and molecular dynamics simulations lasting 100 ns were conducted with GROMACS 2020.6.^57,58^ For the initial setup of the simulation system, the LRRK2-**5** complex was protonated and solvated within a TIP3P water box with a 10 Å size. Buffer ions were strategically added to maintain the overall neutrality of the system. The initial energy minimization phase lasted 1000 ps, after which the system was equilibrated in an isothermal–isobaric (NPT) ensemble under a pressure of 1 bar employing a 2 fs time step and a temperature of 300 K. Additionally, the canonical ensemble NVT was simulated considering the volume (*V*), temperature (*T*), and moles (*N*) for 1 ns. Finally, the root mean square deviation (RMSD), root mean square fluctuation (RMSF), and radius of gyration (*R*_g_) values for the LRRK2-**5** complex system were assessed and compared with those of the apoprotein.

### 2.11 ADMET

The Absorption, Distribution, Metabolism, Excretion, and Toxicity (ADME-Tox)^59,60^ parameters of **5** were systematically evaluated through the Swiss ADME portal (http://www.swissadme.ch/index.php)^61−63^ and the ProTox-II server (https://tox-new.charite.de/protox_II/index.php?site=compound_input).^64^ The c SMILES notation for **5** was input into both platforms for analysis. In addition to standard ADME properties, the “Brain or Intestinal Predicted Permeation” (BOILED-Egg)^62^ method was used to predict its potential to enter the central nervous system (CNS) and to determine whether **5** served as a substrate for *P*-glycoprotein (Pgp), a vital transporter influencing drug absorption and efflux. The absorption potential in the human intestine (HIA) was also explored, considering calculated values for lipophilicity (WlogP) and polarity (TPSA).^65^ This comprehensive analysis provided valuable insights into the pharmacokinetic and toxicological profiles of **5**, aiding in understanding its potential effects within the body.

## 3 Results and Discussion

### 3.1 Synthesis of 5

**Scheme 1:**
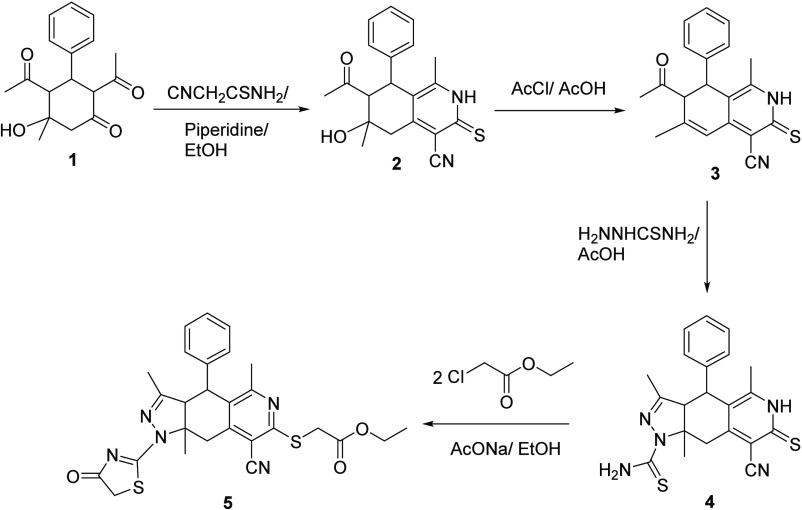
Synthesis Route of **5**

The synthesis of **5** is outlined in Scheme 1. Thus, the reaction of 2-acetylcyclohexanone derivative **1** with 2-cyanothioacetamide in refluxing ethanol in the presence of piperidine as a basic catalyst gave 7-acetyl-8-phenyl-4-cyano-1,6-dimethyl-6-hydroxy-5,6,7,8-tetrahydroisoquinoline-3(2*H*)-thione (**2**). Dehydration of **2** to give 7,8-dihydroisoquinoline **3** was achieved by heating with acetyl chloride in glacial acetic acid at reflux temperature for 2 h. Cyclocondensation of **3** with thiosemicarbazide resulted in the formation of tetrahydropyraoloisoquinoline **4**. Reaction of **4** with a 2 molar ratio of ethyl chloroacetate in refluxing ethanol in the presence of an excess molar amount of anhydrous sodium acetate gave ethyl 2-((8-cyano-3,5,9a-trimethyl-1-(4-oxo-4,5-dihydro-thiazol-2-yl)-4-phenyl-3a,4,9,9a-tetrahydro-1*H*-pyrazolo[3,4-*g*]isoquinolin-7-yl)thio)acetate (**5**) in high yield (83%).

The identities of intermediates **3**(66) and **4**(67) were checked by their IR and NMR spectra that were in agreement with those reported before. The IR spectrum of **5** showed characteristic absorption bands at 3647 and 3496 cm^–1^ for (O–H), 2217 cm^–1^ for (C≡N), 1746 cm^–1^ for (C=O, ester), and 1697 cm^–1^ for (C=O, thiazoline). The ^1^H NMR spectrum of **5** showed the following signals: a multiplet at δ 7.21–7.30 (2H, ArH’s), a doublet at δ 6.90 (2H, ArH’s), a singlet at δ 4.61 (1H, CH), a doublet at δ 4.46 (1H, CH), a quartet at δ 4.16 (2H, OCH_2_ of ester group), a multiplet at δ 3.87–3.98 (3H: SCH_2_ and CH), a multiplet at δ 3.64–3.77 (2H, CH_2_ of thiazoline ring), a singlet at δ 2.40 (CH_3_), a doublet at δ 2.28 (1H, CH), a singlet at δ 2.06 (CH_3_), a singlet at δ 1.77 (2H, H_2_O of crystallization), a singlet at δ 1.60 (CH_3_), and a triplet at δ 1.21 (CH_3_ of ester group). The ^13^C NMR spectrum of **5** displayed the following peaks: δ 187.71, 177.07, 168.91, 160.94, 160.27, 158.89, 149.64, 136.23, 129.50, 127.88, 126.45, 126.37, 113.41, 105.71, 69.86, 64.38, 62.21, 40.50, 38.18, 33.88, 32.62, 26.61, 22.20, 14.82, 14.19, which are in agreement with its structure.

### 3.2 Crystal Structure Description

**Figure 1:**
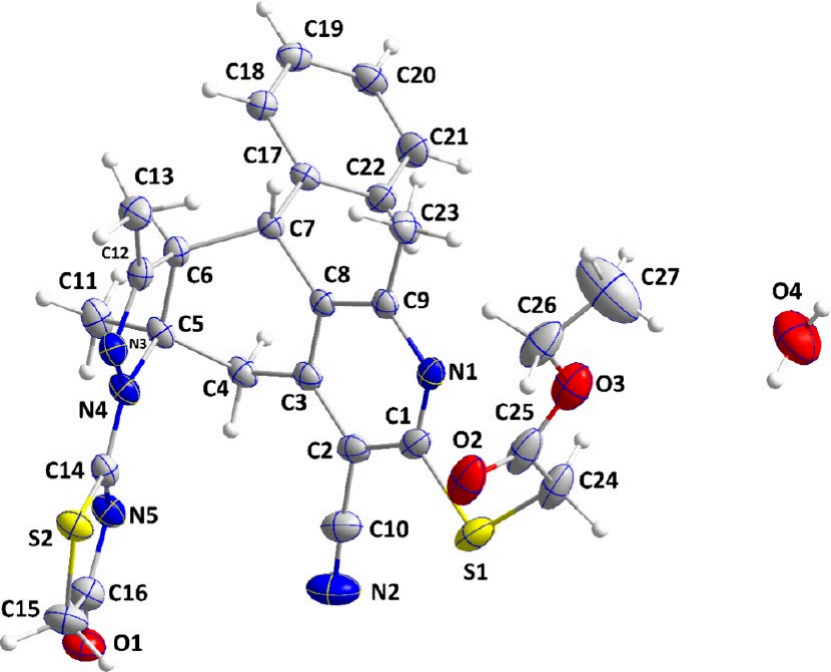
Perspective view of **5** with a labeling scheme and 50% probability ellipsoids. Only the major components of the disorder are shown.

**Figure 2:**
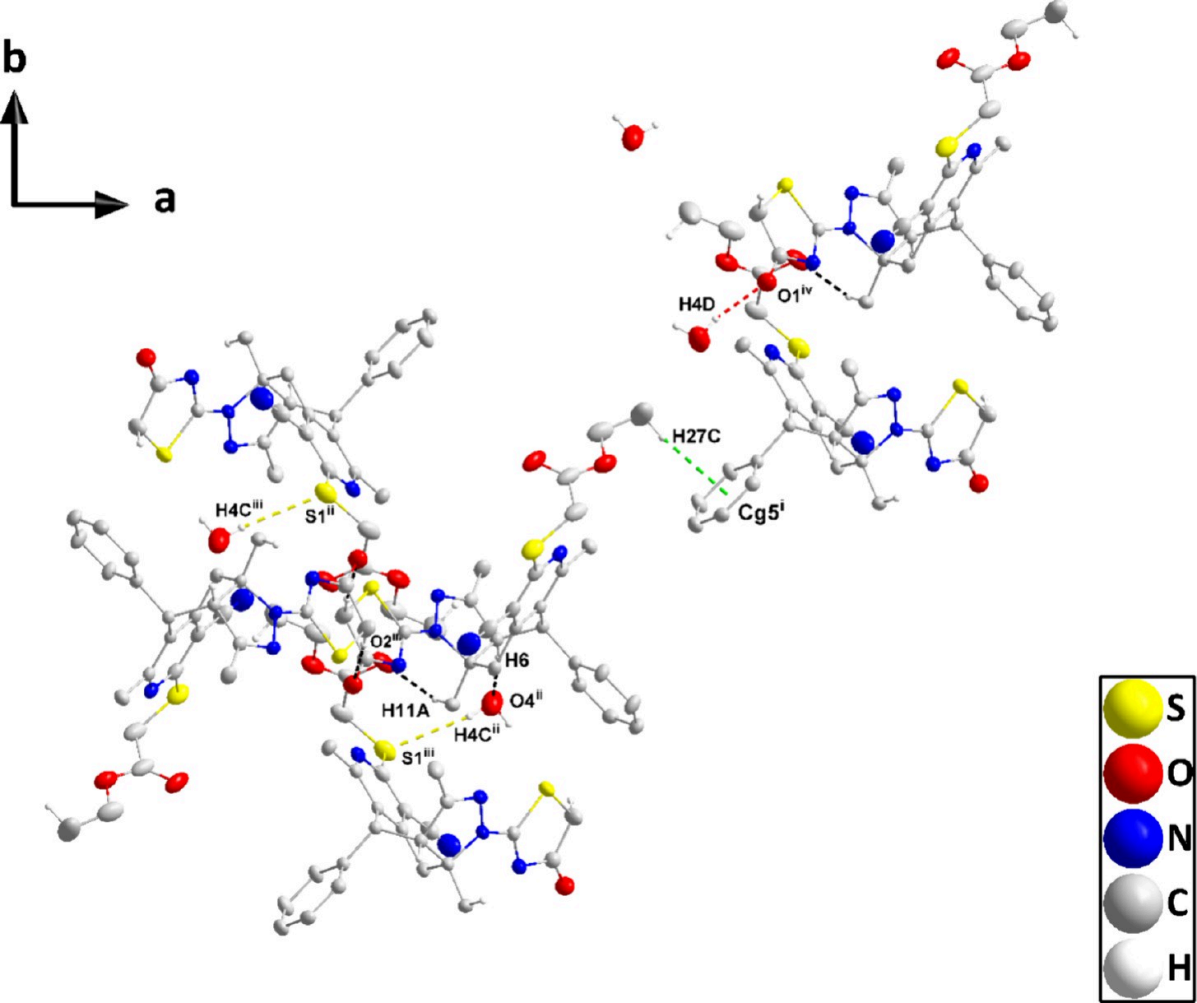
Detail of the intermolecular interactions viewed along the *c*-axis direction. O–H···O, O–H···S, and C–H···O hydrogen bonds are depicted, respectively, by red, yellow, and black dashed lines. The C–H···π(ring) interactions are depicted by green dashed lines. Symmetry codes (i–vi) are given in Table 2, and noninteracting hydrogen atoms are omitted for clarity.

The molecule adopts a “pincer” conformation with the C4···C7 axis of the central ring forming the hinge (Figure 1). A puckering analysis^68^ of the C3···C8 ring gave the parameters *Q* = 0.6630(14) Å, θ = 89.70(12)° and φ = 59.32(17)° with the ring adopting a boat conformation. The dihedral angle between the mean planes of the C17···C32 and the C1/C2/C3/C8/C9/N1 rings is 86.95(4)°, whereas that between the latter and the C5/C6/C12/N3/N4 ring is 69.37(4)°. The dihedral angle between the mean planes of the C5/C6/C12/N3/N4 and C14/N5/C16/C15/S2 rings is 8.31(7)°. In the crystal, thick layers of molecules parallel to (101) and having the ester groups protruding from the faces are formed by C6–H6···O4, C11–H11A···O2, C15–H15A···O1, and O4–H4C···S1 and the corresponding C4–H4D···O1 hydrogen bonds. The layers are linked by inversion-related pairs of C27–H27C···*Cg*5 interactions (Table 1). Figure 2 shows these interactions in portions of two layers viewed approximately edge-on.

**Table 1:** Hydrogen-Bond Geometry (Å, °)

D–H···A	D–H	H···A	D···A	D–H···A	symmetry code
C6–H6···O4^i^	1.00	2.59	3.586(3)	172	(i) *x* – 1/2, – *y* + 3/2, *z* – 1/2
C11–H11A···O2^ii^	0.98	2.44	3.359(2)	157	(ii) −*x* + 1/2, *y* – 1/2, −*z* + 1/2
C15–H15A···O1^iii^	0.99	2.45	3.442(2)	177	(iii) −*x*, −*y* + 1, −*z* + 1
C27–H27C···Cg5^iv^^a^	0.98	2.73	3.706(8)	173	(iv) −*x* + 3/2, *y* + 1/2, −*z* + 1/2
O4–H4C···S1^v^	0.87	2.89	3.691(3)	154	(v) −*x* + 1, −*y* + 2, −*z* + 1
O4–H4D···O1^vi^	0.87	2.04	2.881(3)	164	(vi) *x* + 1, *y* + 1, *z*

a Cg5 is the centroid of the C17···C22 benzene ring.

### 3.3 Hirshfeld Surface Studies

**Figure 3:**
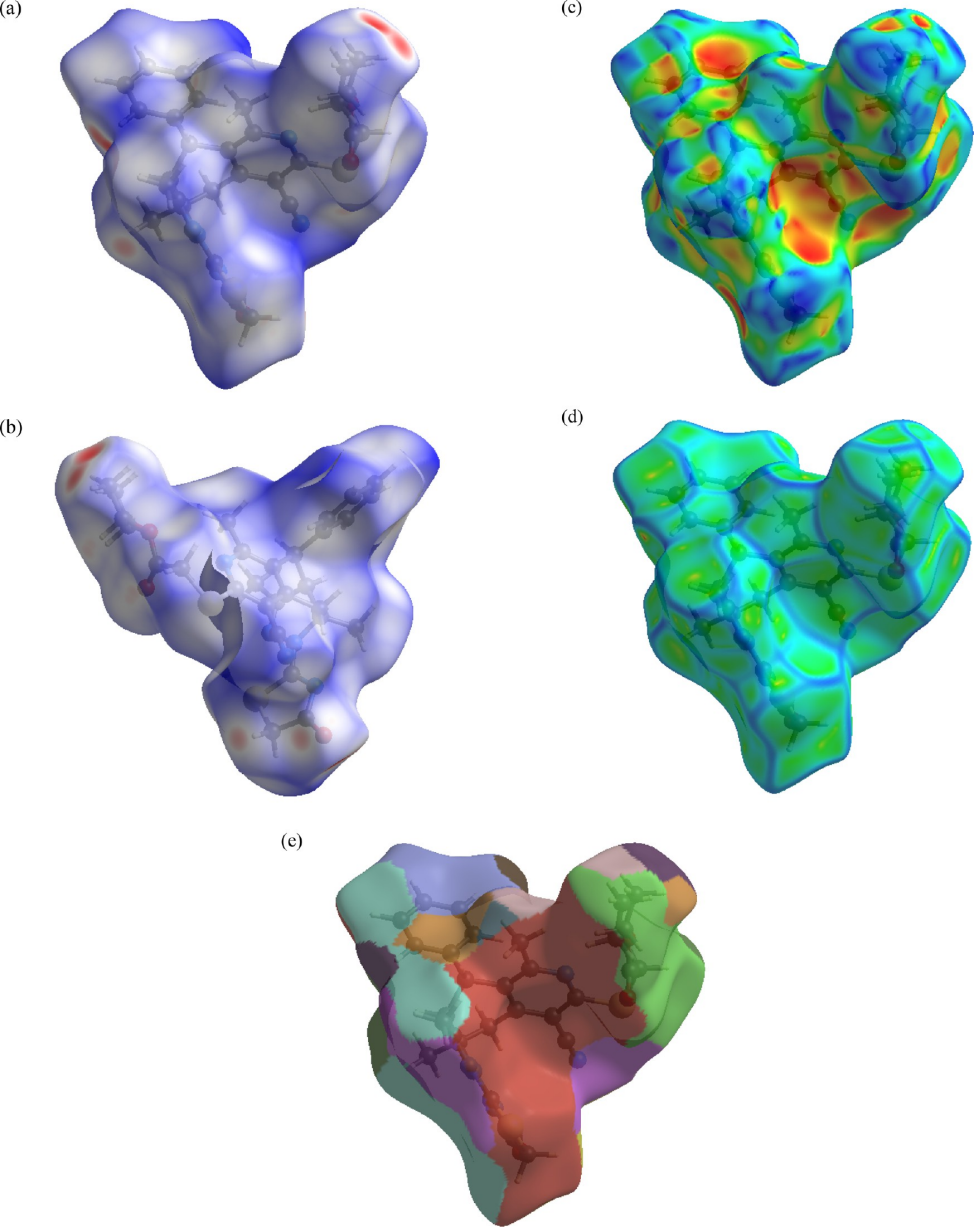
3D HS mapped on (a) *d*_norm_(front), (b) *d*_norm_(back), (c) shape index, (d) curvedness, and (e) fragment patch.

The minimum, maximum, and mean values of the Hirshfeld surface (*d*_norm,_ shape index, curvedness, and fragment patch) are tabulated in Table S2. Figure 3 shows the standard resolution molecular HS (*d*_norm,_ shape index, curvedness, and fragment patch) for **5**.

Detailed descriptions of these surfaces and their interpretation have been published.^25,27,69−76^ Accordingly, the HS of **5** shows that intermolecular hydrogen bonds play more important roles in the crystal packing than C–H···π(ring) interactions.

The 2D fingerprint plots indicate which interaction plays the most important role in intermolecular hydrogen bonding.

**Figure 4:**
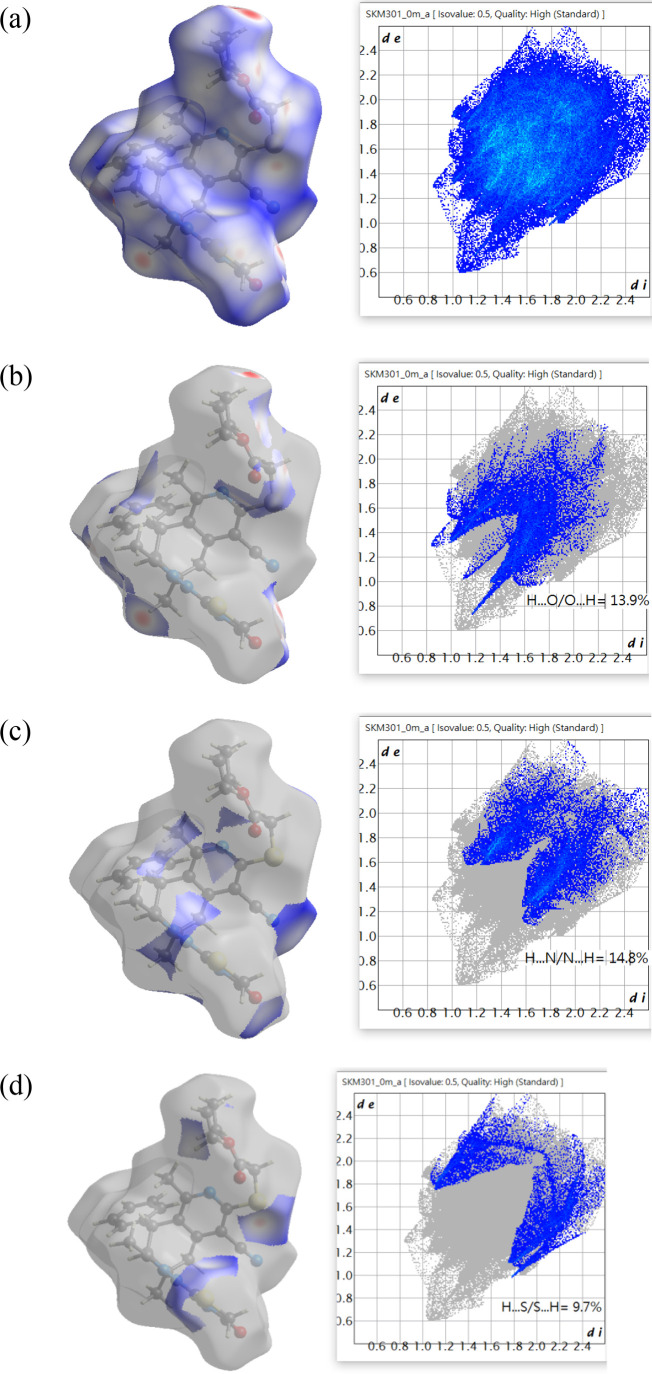
Two-dimensional fingerprint plots for **5** (a) full, (b) resolved by the O···H/H···O contacts, (**c**) resolved by the N···H/H···N contacts, and (d) resolved by the S···H/H···S contacts.

**Figure 5:**
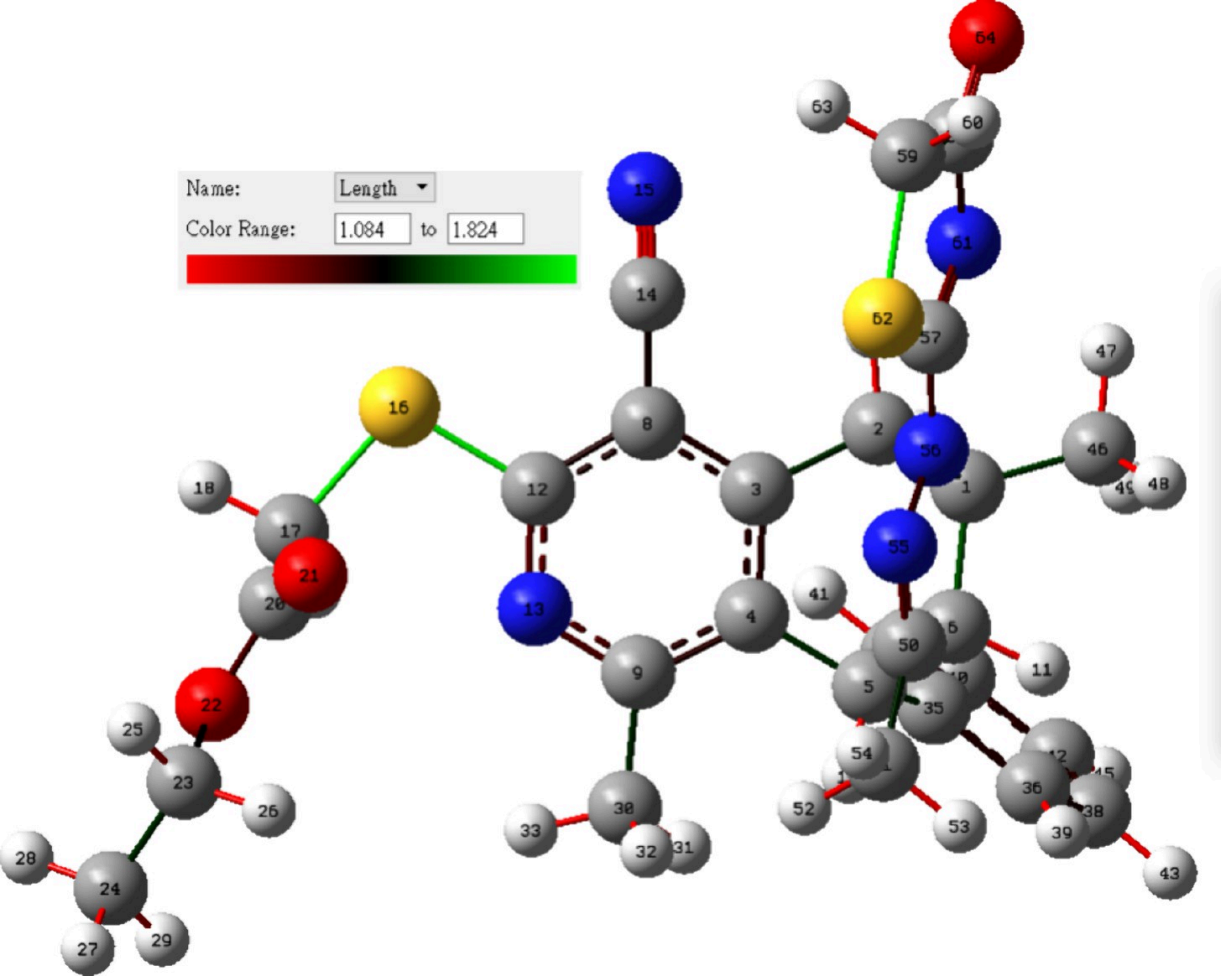
B3LYP-optimized geometry of **5** (bond lengths in Å).

Of these, the largest contributor is the N···H/H···N contact with the ···H/H···O and S···H/H···S contacts close behind (Figure 4 and Table S3). However, despite the N···H/H···N contacts making the largest contribution, the tips of the two “spikes” in Figure 4c are broad and at minimum are at *d*_e_ + *d*_i_ ≈ 2.8 Å, which is comparable to the sum of the van der Waals radii of H and N (2.75 Å). This indicates that these contacts represent weak attractive interactions at best and so likely contribute little to the lattice energy of the crystal. Note also that there are no *specific* N···H interactions listed in Table 1 and the shortest contact identified by *PLATON CALCALL*(70) is C23–H23C···N5^i^ (symmetry code: (i) 1/2 – *x*, 1/2 + *y*, 1/2 – *z*) with N···H = 2.81 Å, which is 0.06 Å greater than the sum of the van der Waals radii. Figure 5b is interesting in that the “spikes” vary significantly in sharpness and the whole pattern is not fully symmetric. This is the result of the O···H interactions coming from both the −H···O and C–H···O hydrogen bonds in which the O···H distances are significantly different (Table 1).

### 3.4 DFT Studies

A gas-phase DFT study was performed using the B3LYP functional to rationalize the relationship between the intrinsic electronic properties, chemical reactivities, and biological activities of **5** with the B3LYP-optimized geometry depicted in Figure 5.

**Figure 6:**
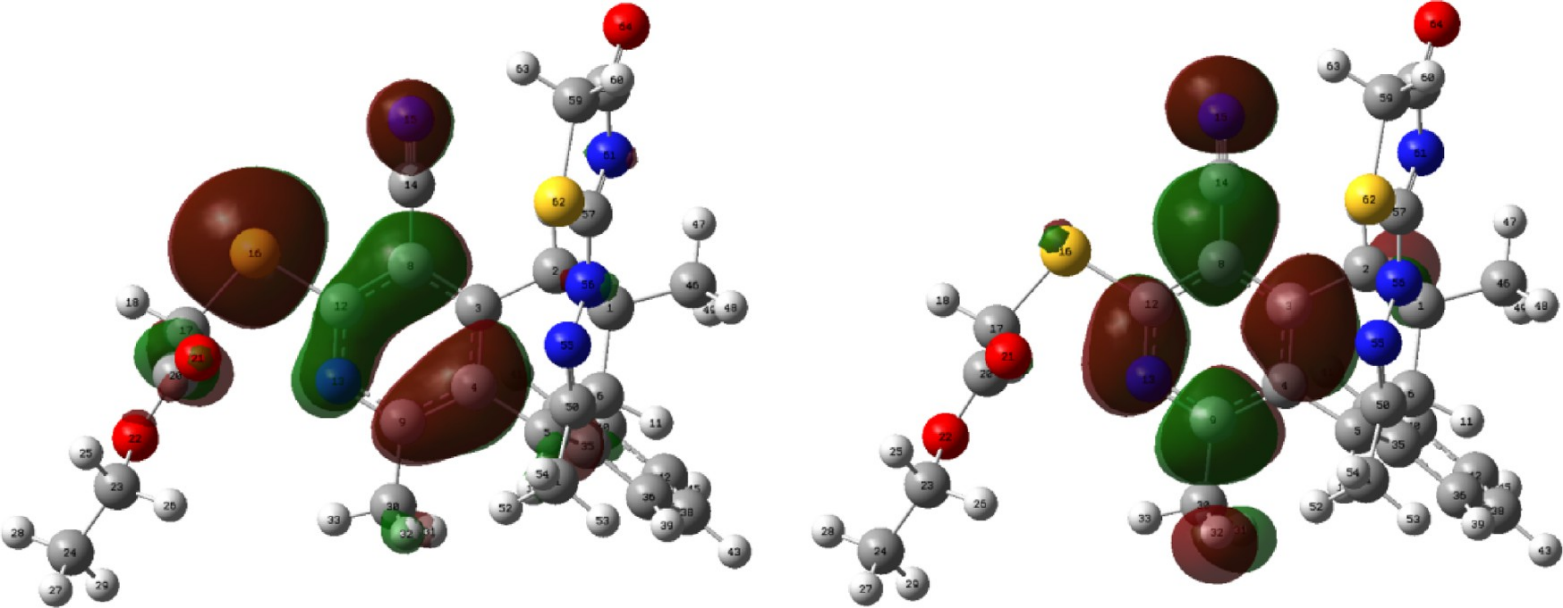
HOMOs and LUMOs of **5** (isovalue = 0.02 au).

According to frontier molecular orbital theory, one can determine a molecule’s nucleophilicity or electrophilicity by focusing on the highest occupied and lowest unoccupied molecular orbitals (HOMO and LUMO). Thus, one evaluates the localization of the HOMO orbital because electrons from this orbital have the best probability of participating in a nucleophilic attack, whereas a site in LUMO is a suitable electrophilic site. The frontier molecular orbitals for **5** are shown in Figure 6 where it appears that the transition from the HOMO to the LUMO mixes the ππ* transition with the nπ* transition.

**Figure 7:**
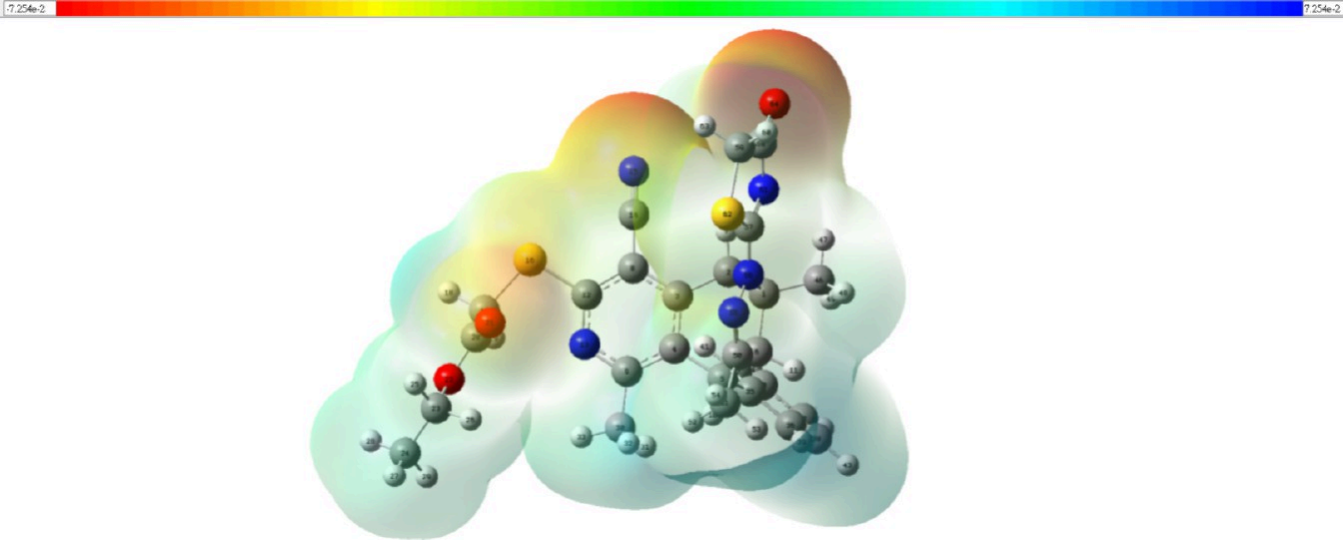
MEP of **5** (isovalue = 0.0004 a.u).

Molecular electrostatic potentials (MEPs) are assessments of the strength of interactions among neighboring charges, nuclei, and electrons at a specific point, allowing charge distribution and charge-related features of molecules to be examined with the results presented graphically in Figure 7. Electrophiles may be attracted to the red-colored region because it represents the lowest electrostatic potential. Blue, on the other hand, has the largest electrical potential and may be attractive to nucleophiles. The entire electron density is calculated using the full density matrix, and the resulting MEP is mapped on its surface. From its MEPs, the nitrogen and oxygen atoms are expected to be nucleophilic sites in **5** in terms of their electronegativities.

#### 3.4.1 The TDDFT Results

**Scheme 2:**
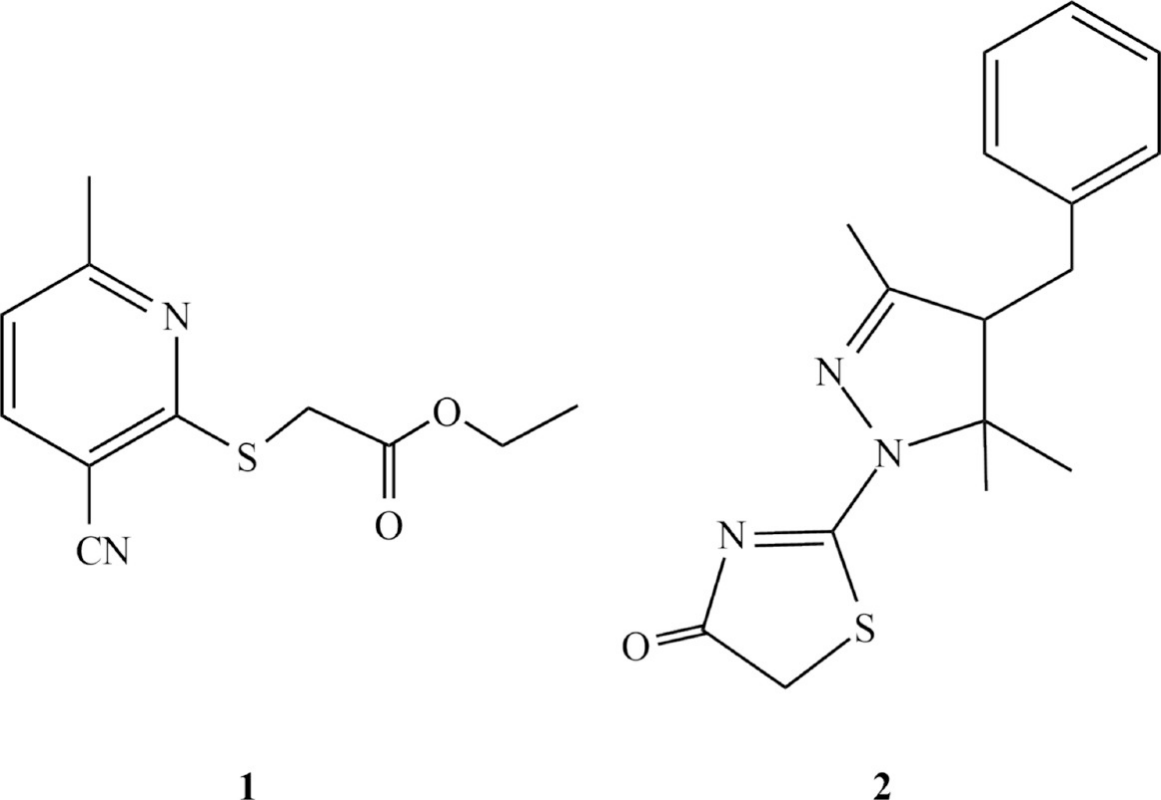
Fragmentation of **5**

**Figure 8:**
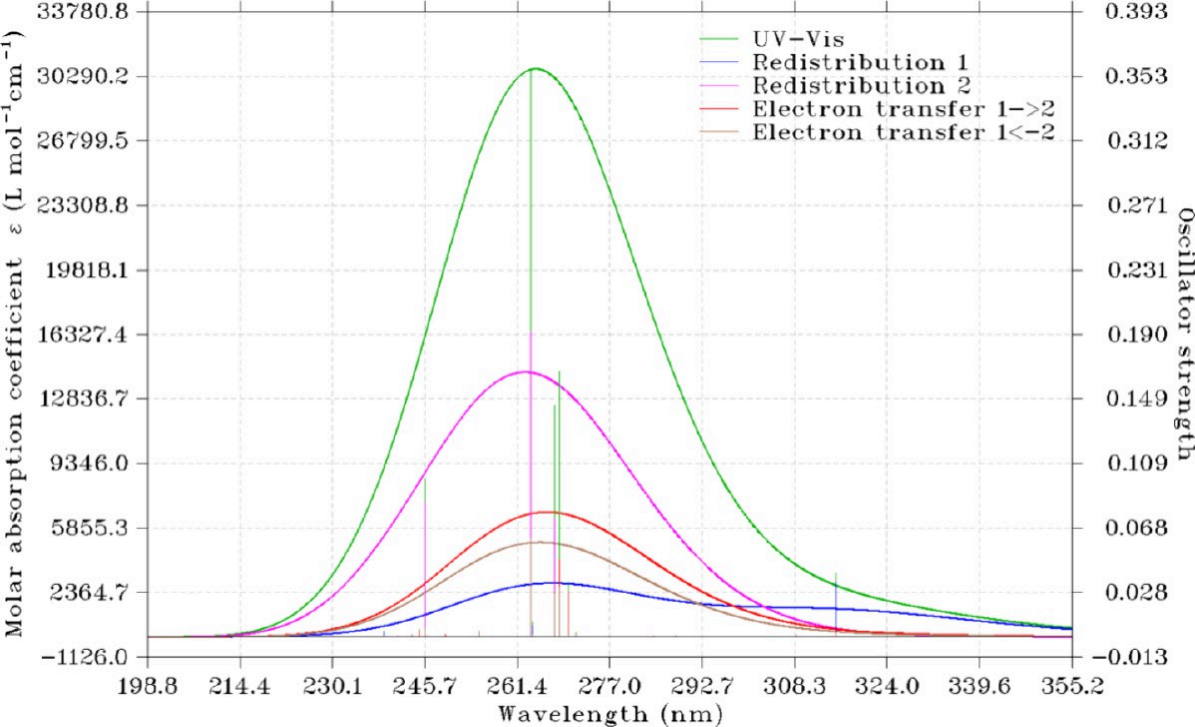
Charge transfer spectrum of the molecule studied.

**Figure 9:**
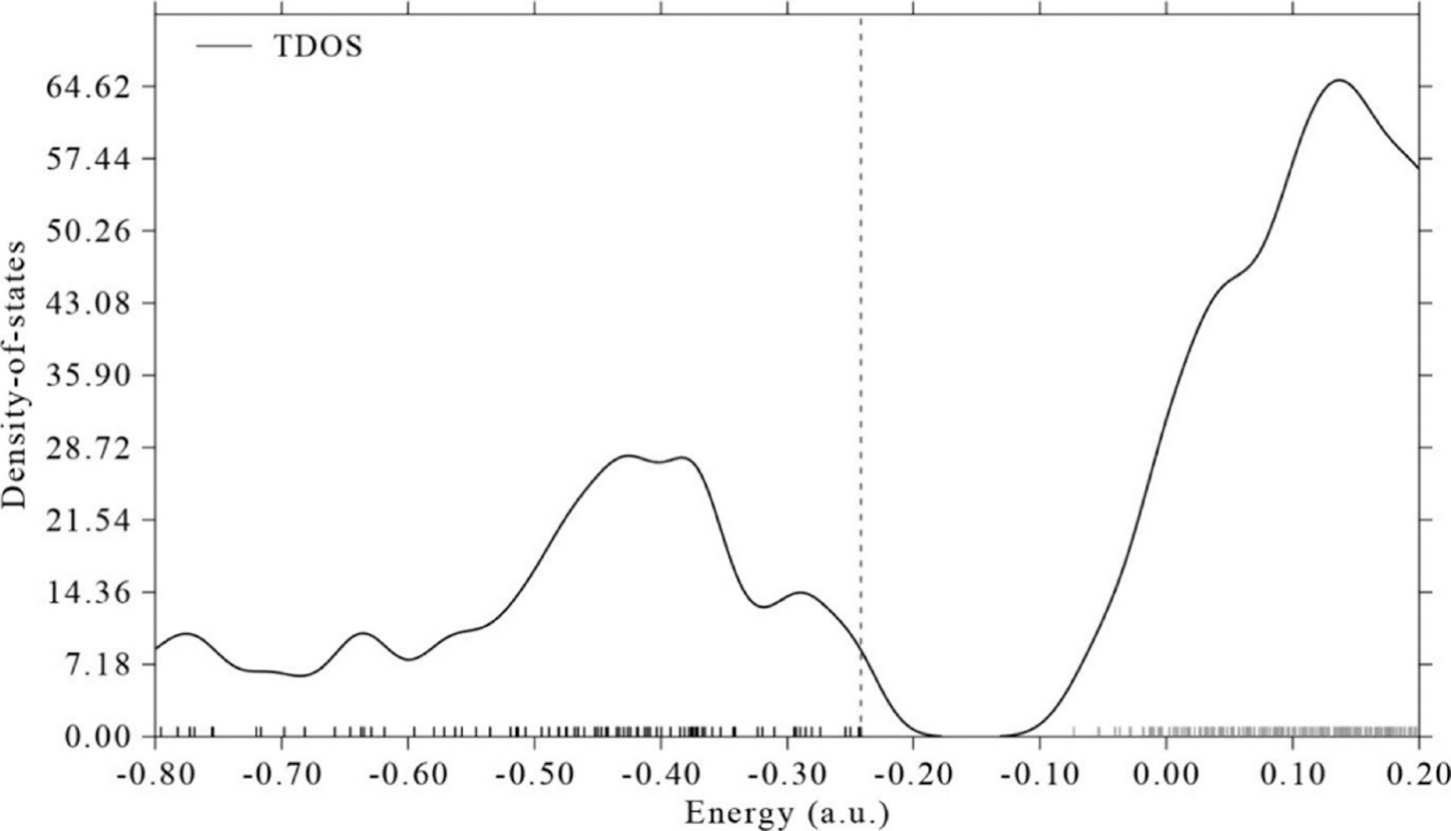
TDOS spectrum of **5**.

In this study, the 20 lowest singlet states of the compound studied are investigated using the TDB3LYP/6-311++G** theoretical level. Moreover, to study whether the intrafragment electron redistribution or charge transfer between fragments is more critical for the electronic transition of the compound under study, we divided it into two fragments (Scheme 2). The calculated wavelength, oscillator strength, and orbital contributions and the degree of charge transfer of the respective electronic transition for **5** are summarized as Table S5 in the Supporting Information. The electronic transition with the largest oscillator strength is S_0_ → S_11_. The charge transfer (CT) spectrum of the titled compound calculated by the Hirshfeld method is plotted using the Multiwfn program and depicted as Figure 8.^77^ As depicted in Figure 8, there is no pure charge transfer excitation for **5**. The electron redistribution within fragment 2 plays a dominant role in the CT spectrum of **5**. Furthermore, the charge transfer from fragment 1 to fragment 2 plays a more important role in the CT spectrum of **5** than that from fragment 2 to fragment 1. Furthermore, the total density of state (TDOS) spectrum of **5** is also generated and plotted as Figure 9 shows using the Multiwfn program.^35^ The vertical dashed line in Figure 9 indicates the location of the HOMO of the investigated molecule.

#### 3.4.2 The QTAIM analysis

**Figure 10:**
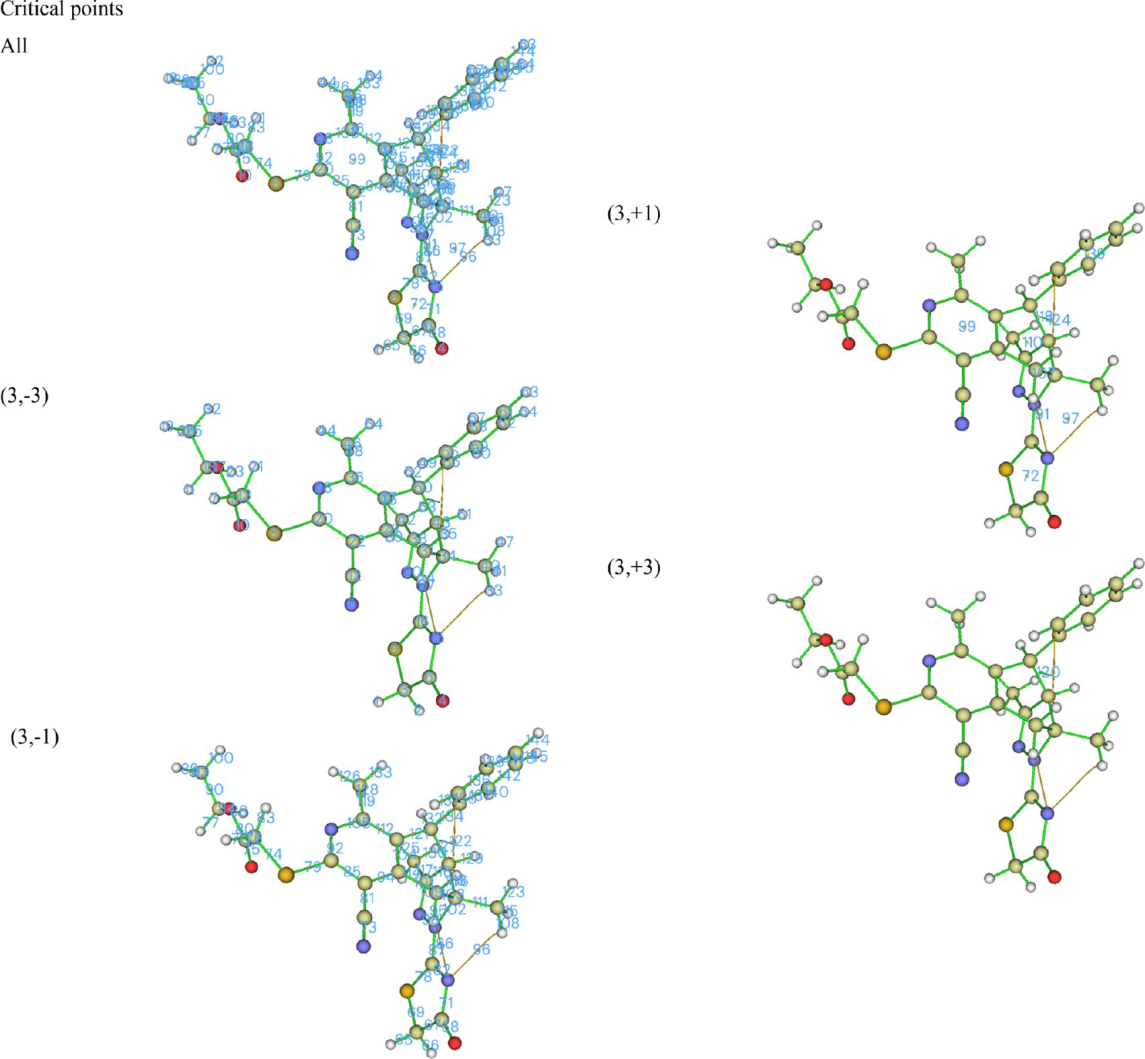
Critical points of **5**.

Under the condition that the Poincare–Hopf relationship is satisfied, there are 145 calculated critical points (CPs) for **5**, and of these, there are 64, 71, 9, and 1 for the (3,–3), (3,–1), (3,+1), and (3,+3) CPs, respectively (Figure 10). All the (3,–3) CPs correspond to the atomic nuclei of the molecule, whereas the (3,–1) CPs are located between two atoms forming a chemical bond except points 86, 96, and 121. All the (3,+1) CPs correspond to ring critical points, and one (3,+3) CP corresponds to a local minimum with electron density rising in all three directions of space indicating it to be a cage critical point that is found when several rings form a cage. The critical points designated by 86, 96, and 122 indicate that noncovalent interactions exist between H7 and N61, H47 and N61, and H34 and C37, respectively (Figure 5). A QTAIM study is a powerful tool to investigate intra- or intermolecular hydrogen bonding of several systems.^78−85^ According to QTAIM, if D–H forms a hydrogen bond with A, there should be a CP between H and A. In addition, criteria about the electron density (ρ_b_) and the Laplacian of electron density (∇^2^ρ_b_) at CPs have been established by Koch and Popelier to distinguish hydrogen bonding from van der Waals interactions.^86^ By this criterion, a hydrogen bond is formed if the BCP between H and A has an electron density in the range of 0.002–0.034 au and a Laplacian of the electron density in the range of 0.024–0.139 au. BCP 86, 96, and 122 have an electron density of 1.17 × 10^–2^, 1.14 × 10^–2^, and 6.62 × 10^–3^ a.u., respectively_._ Furthermore, they have a Laplacian of the electron density of 3.80 × 10^–2^, 3.74 × 10^–2^, and 2.19 × 10^–2^ a.u., respectively_._ Although H7 and N61, and H47 and N61 undoubtedly form a hydrogen bond, whether H34 and C37 form a hydrogen bond needs further investigation. Moreover, Liu and co-workers have established a relationship between the hydrogen bonding strength (BE) and the electron density (ρ_b_) at the CP corresponding to the hydrogen bond, which is expressed by eq 8.^87^
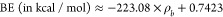
8

Using this approximation, the strengths of the hydrogen bond between H7 and N61 and H47 and N61 are −1.86 and −1.81, respectively, which agree with the points made in Section 3.3 that all H···N interactions are quite weak.

#### 3.4.3 The NCI Study

**Figure 11:**
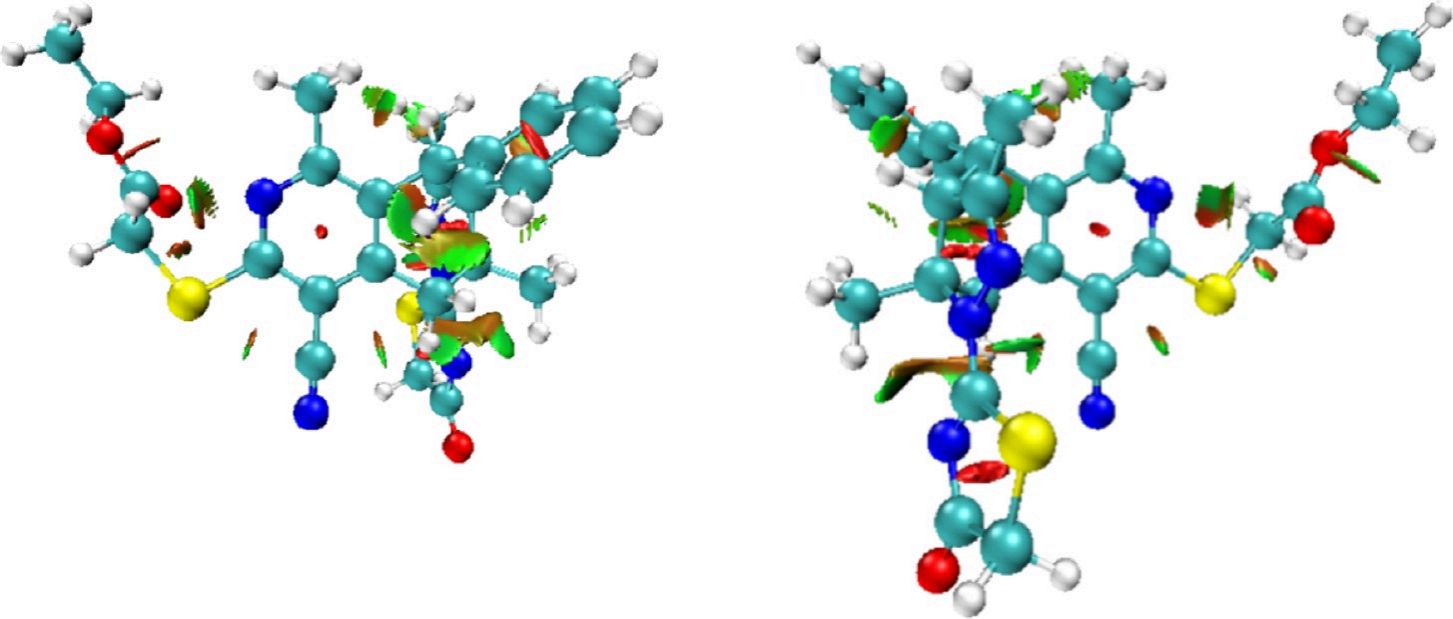
NCI isosurfaces of **5** (right: rotation by 180°).

As presented, whether H34 and C37 form a hydrogen bond needs further investigation. Therefore, the NCI was further performed to study the intramolecular noncovalent interactions within **5**. To obtain as much as possible all noncovalent interactions present in **5**, a high-quality grid is used in this study. The resulting intramolecular noncovalent interactions in **5** are depicted in Figure 11 and Figure S4. The default RDG isosurface is 0.5, and the color range is −0.035 to 0.02. Different types of ranges in the color-filled RDG isosurfaces are simply identified by examining their colors. The bluer region implies the stronger attractive interaction; in contrast, the interaction region marked by the green circle can be identified as the vdW interaction region. Moreover, regions filled with red correspond to strong steric interaction. According to the no blue-filled areas in the NCI plot drawn by the VMD program^88^ (Figure 11), the molecule under study should not have intramolecular hydrogen bonds, only some van der Waals forces. Significantly, the NCI study shows a different result from the QTAIM study, which shows that two intramolecular hydrogen bonds, C2–H7···N61 and C46–H47···N61, should exist in **5**.

#### 3.4.4 The Result of the NBO Analysis

Because there is a difference between the result of the QTAIM analysis and that of the NCI study, the second-order perturbation theory was used to investigate the intramolecular hydrogen bonding in **5** to compare with the results of NCI and QTAIM calculations. When a hydrogen bond occurs, the nonbonding orbital of the hydrogen-bonded acceptor (n_A_) and the antibonding orbital of the H···donor bond (σ_H-D_*) should interact orbitally. As a result of this orbital interaction, the H···D bond strength and bond order should be reduced and decreased, respectively. In our previous study, this orbital interaction was used to investigate the strongest hydrogen bond among several heterocyclic rings,^89−91^ and the correlation between the orbital interaction of n_N_ with σ_H–C_* and the strength of a hydrogen bond was investigated to see whether or not a hydrogen bond formed between C–H and N. For **5**, the interaction between a lone pair and the X–H antibonding orbital basing on the second-order perturbation theory is summarized in Table 2. The interaction energy between orbitals is so tiny that it is impossible to determine whether intramolecular hydrogen bonds exist in the molecule under study.

**Table 2:** NBO Results for **5**

the type of n_A_	the electron configuration of n_A_	the type of orbital interaction	the interaction energy (in kcal/mol)^b^	the occupancy of σ_H-D_*	the bond order of σ_H-D_^c^
LP(N13)^a^	s(26.72%)p(73.22%)d(0.06%)	LP(N13) -σ*(C17–H18)^a^	0.72	0.01108	0.7820
LP(N13)^a^	s(26.72%)p(73.22%)d(0.06%)	LP(N13) -σ*(C17–H19)^a^	0.03	0.01793	0.7806
LP(N61)^a^	s(32.28%)p(67.65%)d(0.07%)	LP(N61) -σ*(C46–H47)^a^	0.55	0.00715	0.7812
LP(N61)^a^	s(32.28%)p(67.65%)d(0.07%)	LP(N61) -σ*(C2–H7)^a^	0.76	0.01375	0.7721

a Please see the atomic designations in Figure 5.

b The interaction energy was calculated based on the second-order perturbation theory.

c The listed values are the atom–atom overlap-weighted NAO bond order.

#### 3.4.5 The CDFT Study

**Figure 12:**
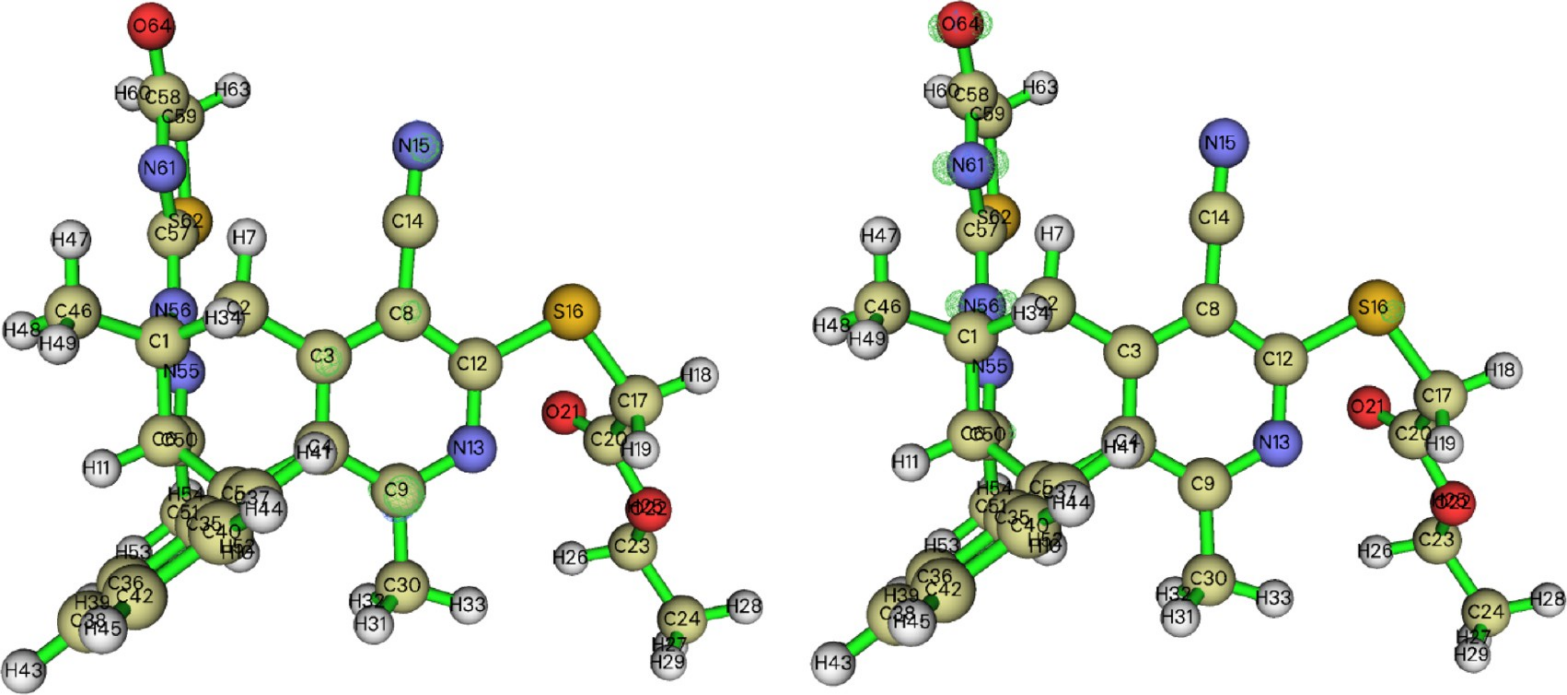
Fukui functions for **5** (isosurface value = 0.01).

Using the high-quality grid, the Fukui function (*f*^+^(*r*) and *f*^–^(*r*)) of **5** is visualized by mesh as Figure 12 shows. The local electrophilicity (ω_L_) of a specific atom can be represented by the *f*^+^(*r*) Fukui function, whereas the local nucleophilicity (N_L_) of a specific atom can be represented by the *f*^–^(*r*) Fukui function. Their relationship is described as
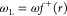
9
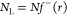
10where ω and *N* represent the global electrophilicity and the global nucleophilicity, respectively.

For obtaining the global reactivity descriptors for **5**, single-point energy calculations are done on the cationic and anionic radical of **5**. Then the ionization energy and electron affinity of **5** are calculated based on the following equations:
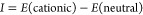
11
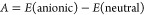
12

Accordingly, the global reactivity descriptors for **5**, i.e., chemical potential (μ), electronegativity (χ), chemical hardness (η), softness (*S*), electrophilicity (ω), and nucleophilicity (*N*), are summarized in Table 3. It is noteworthy that η is not equal to the energy gap (Δ*E*_g_) between HOMO and LUMO (4.585 eV). Based on Koopmans’ theorem, *I* = −E_HOMO_ = 6.572 eV and *A* = −*E*_LUMO_ = 1.987 eV; therefore, η should be equal to Δ*E*_g_. It indicates that *I* and *A* both show deviations from −*E*_HOMO_ and −*E*_LUMO_.

**Table 3:** Selected Electronic Properties for **5**

	5
*I* (in eV)	7.8056
*A* (in eV)	0.6397
μ (in eV)	–4.2226
χ (in eV)	4.2226
η (in eV)	7.1659
*S* (in (eV)^−1^)	0.1395
ω (in eV)	1.2441
*N* (in eV)	2.5458

The smaller (greater) value of hardness (softness) a molecule has, the more reactive it should be. Therefore, from Table 3, **5** may be a stable molecule. Furthermore, there are previous relevant studies^92,93^ showing that the toxicity of a species seems to have a relationship with its electrophilicity index (ω). From Table 3, **5** should have a low toxicity due to its large hardness and small softness.

#### 3.4.6 The ALIE Study

**Figure 13:**
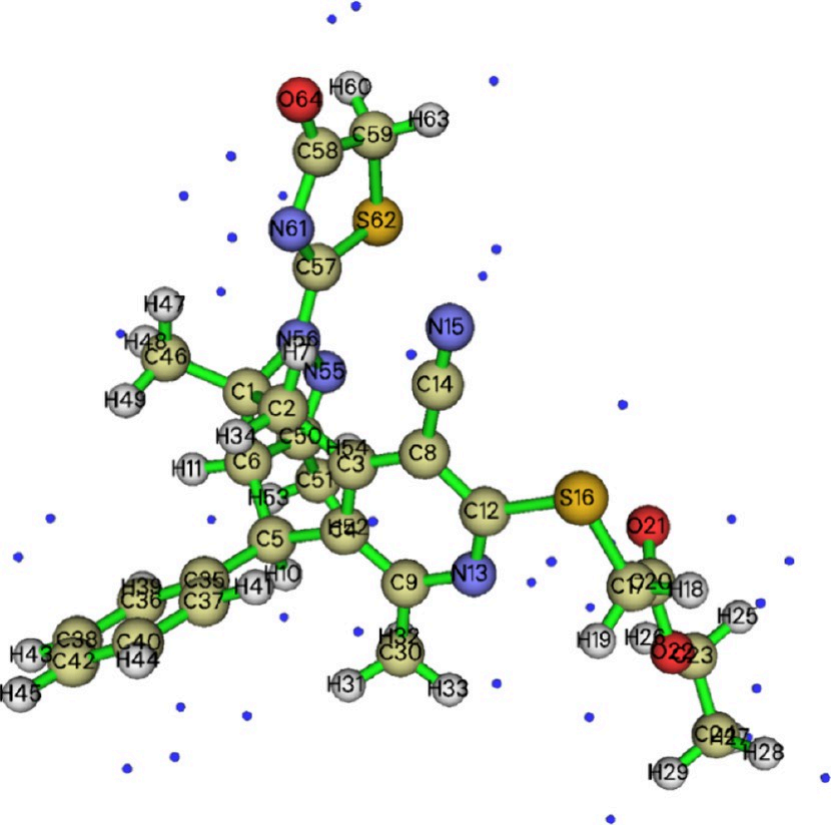
Result of the ALIE analysis of **5**.

In this study, the site with the minimum value of the ALIE was represented as a blue dot and plotted in Figure 13 where there are 39 positions with a minimum value of ALIE. These positions should be the reactive centers with electrophiles or free radicals.

### 3.5 Molecular Docking Study

**Figure 14:**
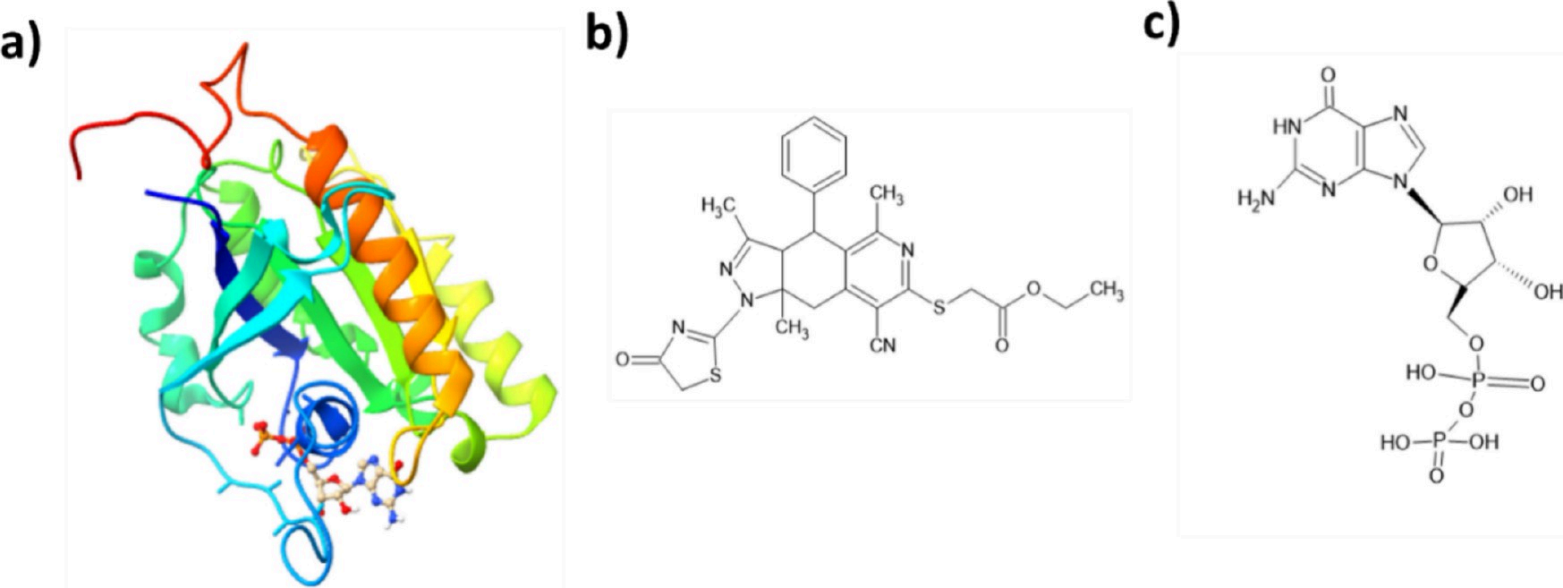
(a) The 3D structure of the LRRK2 protein and the 2D structure of (b) **5** and (c) the LRRK2-native ligand.

Molecular docking is a computational technique illustrating ligand affinity for protein receptors.^94^ The 3D structure of the LRRK2 protein (a) and the 2D structure of **5** (b) and the native ligand of the protein (c) are shown in Figure 14. The binding energies for **5** and the native ligand with LRRK2 were calculated as −3.34 and −8.10 kcal/mol, respectively.

**Figure 15:**
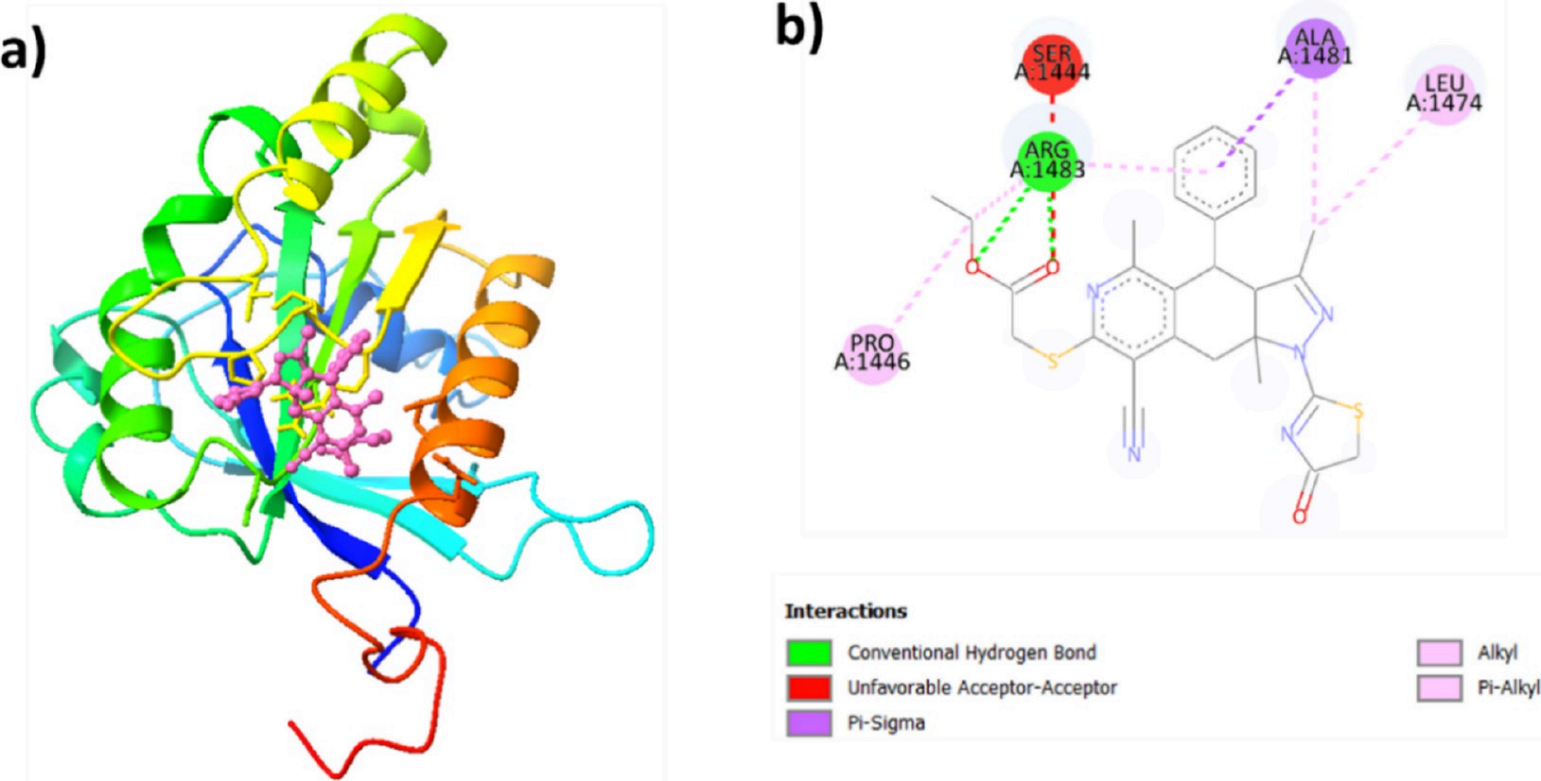
(a) The best-docked pose of the LRRK2-**5** complex and (b) its ligand interaction plot

The interaction plot (Figure 15) shows that **5** forms two conventional hydrogen bonds with ARG A:1483; five alkyl bonds with ARG A:1483, LEU A:1474, ALA A:1481, and PRO A:1446; and a pi−σ bond with ALA A:1481, all of which contribute to the stabilization of the protein-**5** complex. However, there are also two unfavorable acceptor–acceptor bonds with SER A:1444 and ARG A:1483 indicating a less favorable interaction.

**Figure 16:**
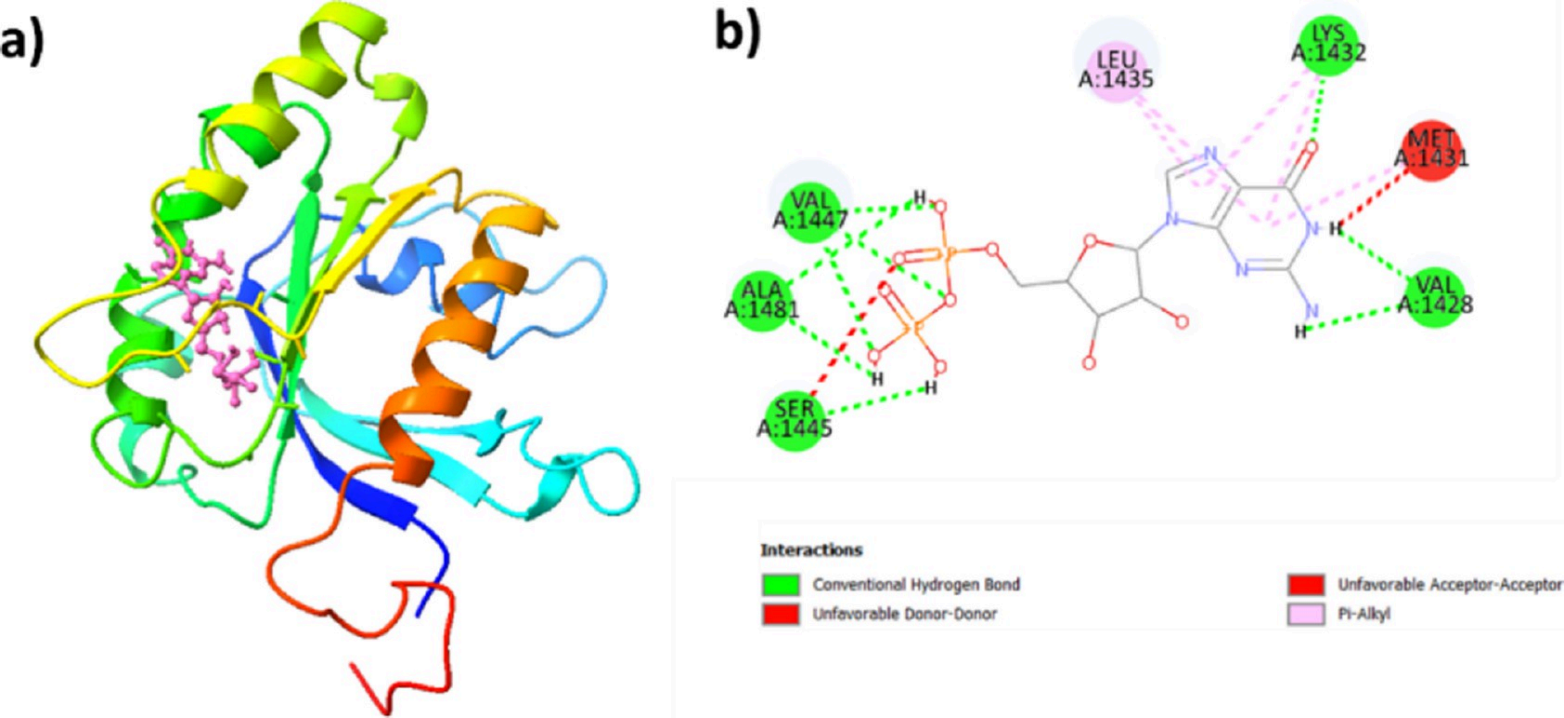
(a) The best-docked pose of LRRK2-native ligand complex and (b) its ligand interaction plot.

As shown in Figure 16a, the native ligand forms three conventional hydrogen bonds with VAL A:1447, two with ALA A:1481 and VAL A:1428, and one with SER A:1445 and LYS A:1432,. Additionally, it forms five alkyl bonds, with two of them specifically interacting with LEU A:1435 and LYS A:1432, and one bond with MET A:1431. All of these contribute to the stability of the protein–ligand complex, but there are two unfavorable donor–donor bonds with SER A:1445 and VAL A:1428. Overall, **5** shows a propensity for favorable bonding patterns, highlighting the potential advantages in optimizing its design for good binding affinity. Table S4 describes interactions within the LRRK2-**5** complex and the LRRK2-native ligand complex.

### 3.6 Molecular Dynamics

A molecular dynamics (MD) simulation^95−98^ was conducted for a duration of 100 ns, and the results were evaluated to explore the structural and conformational changes during the formation of the LRRK2-**5** complex.

**Figure 17:**
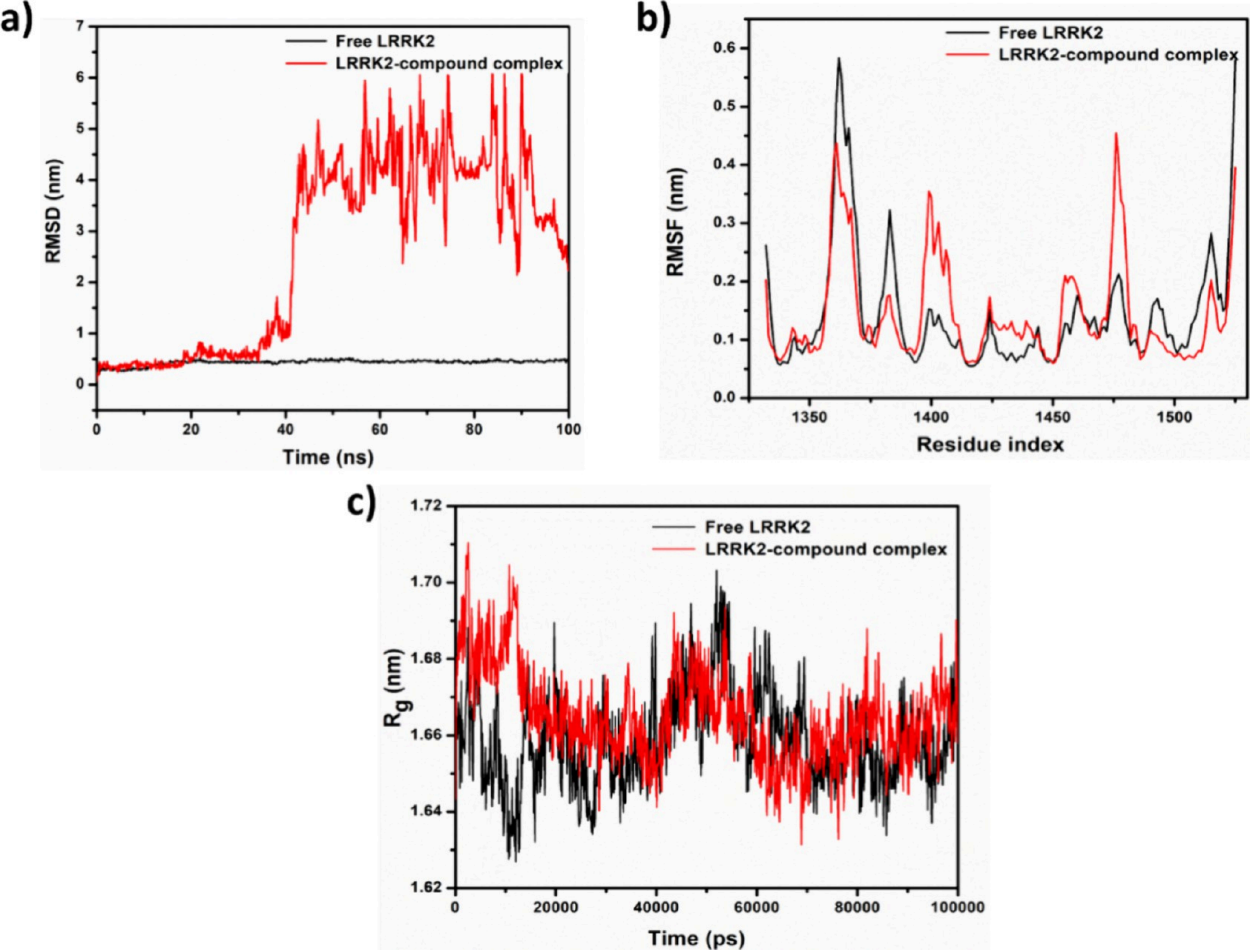
(a) The root mean square deviation (RMSD), (b) root mean square fluctuations (RMSF), and (c) radius of gyration (*R*_g_) for free LRRK2 and the LRRK2-**5** complex.

RMSD is a measure of the average deviation of the positions of atoms from a reference position over time. Figure 17a shows the RMSD plot generated for both free LRRK2 and the LRRK2-**5** complex over a simulation period of 0 to 100 ns. The complex appears stable up to 35 ns and maintains structural consistency, but after 35 ns, a noticeable deviation is observed, indicating a dynamic conformational change in the complex system compared to the free LRRK2 protein. Another significant deviation occurs between 44 and 94 ns, after which the system gradually reaches an approximately 2 nm deviation, close to 100 ns. The observed deviations suggest dynamic structural alterations in the complex throughout the 100 ns simulation period.

RMSF is the measure of the average fluctuation of atomic positions in a molecular system over time. Figure 17b shows the RMSF of free LRRK2 and the LRRK2-**5** complex. The RMSF values for the LRRK2-**5** complex system at residues 1361 and 1383 are lower than those for the free LRRK2 protein, whereas at residues 1401 and 1477, the reverse is true (0.35 nm for complex and 0.14 nm for free ligand and 0.43 vs 0.19 nm, respectively). This indicates reduced fluctuation in the first two regions of the complex compared to the corresponding regions in the free protein and greater fluctuation for the complex compared to the free protein in the latter two regions. Between residues 1401 and 1477, only minor fluctuations are noted in both. The results indicate that the binding of **5** to the protein stabilizes that portion around residues 1361 and 1383 while causing the regions of the protein around residues 1401 and 1477 to become more flexible and have minimal effects on the portion between residues 1401 and 1477.

The compactness of a protein molecule can be measured using the radius of gyration, *R*_g_, which was determined for 0 to 10,000 ps to estimate the conformational alterations and compactness of the protein-**5** complex (Figure 16c). From 0 to 18047 ps, the complex system had higher *R*_g_ values than those of the free LRRK2 protein, indicating that the protein was unfolding. After that, up to 61504 ns, the *R*_g_ values of the complex system became stable, suggesting that the protein had reached a more extended conformation. After this point, there were no significant changes in the *R*_g_ plot between the protein and the complex. This suggests that the protein had reached a stable conformation in the presence of **5**. The stability of the complex system could be due to the binding of the ligand to the protein, which may have prevented further unfolding of the protein.

### 3.7 ADMET

The term “drug-likeness” denotes the delicate balance between various molecular properties and structural features essential for the development and discovery of pharmaceuticals. Lipinski’s five rules play a pivotal role in contributing to drug discovery, particularly in evaluating the bioavailability of bulk materials and assessing their drug-like characteristics during the development of a specific compound.^99,100^Table 4 provides information about the molecular properties of **5**, its druglikeness (Lipinski and Veber), as well as its solubility, absorption, and distribution characteristics. The molecular weight (MW) and MlogP values suggest moderate lipophilicity. It features seven hydrogen bond acceptors (HBA), zero hydrogen bond donor (HBD), a TPSA of 158.61, and seven rotatable bonds (Nrot). As it is poorly soluble, it would not be easily absorbed as a drug. Additionally, the WlogP and GIA values, low Pgp, and absence of penetration of the blood–brain barrier (BBB) present additional problems for it to function as an effective drug.

**Table 4:** Estimated “Drug-like” Characteristics as well as the Compound’s Estimated Absorption and Distribution^a^

compound	Lipinski drug like Veber drug like					solubility		ABS absorption & distribution			
MW g/mol	MlogP	HBA	HBD	TPSA	Nrot	class (ESOL)		WlogP	GIA	Pgp	BBB
533.66	2.72	7	0	158.61	7	poorly soluble	–7.71	3.16	Low	Yes	No

a ABS (A bioavailability score in >10% F), method used in the Lipinski drug-like criteria; MW, molecular weight in g/mol; HBA, hydrogen bond acceptors; TPSA, topological polar surface area in Å^2^; GIA, gastrointestinal absorption; BBB, blood–brain barrier permeation; Nrot, number of rotatable bonds; Pgp, *P*-glycoprotein substrate; HBD, hydrogen bond donors; and WlogP, partitioning coefficient calculated by Wildman et al., the method used in BOILED-Egg.

Table 5 shows the metabolism and excretion, as well as toxicity end points, of CYP450 inhibitors for different enzymes. CYP450 enzymes play a crucial role in drug metabolism, and their inhibition can lead to drug toxicity. This information shows whether the CYP450 inhibitors interact with different enzymes (1A2, 2C19, 2C9, 2D6, and 3A4). The toxicity end points include hepato, carcino, immuno, mutagen, and cyto. The values in the table represent the strength of inhibition for each enzyme and toxicity end point, with higher values indicating stronger inhibition. For example, 2C19 and 2C9 are strong inhibitors of carcino, whereas 3A4 is a weak inhibitor of mutagen. CYP450-mediated metabolism is the major route of elimination for many drugs, and inhibition of these enzymes can lead to drug accumulation or decreased drug metabolism, potentially resulting in clinical toxicity or enhanced drug effects.

**Table 5:** Excretion and Metabolism Inhibition by CYP450 Isoenzymes and Toxicity End Points of **5**

metabolism and excretion CYP450 inhibitors					toxicity end points				
1A2	2C19	2C9	2D6	3A4	hepato	carcino	immuno	mutagen	cyto
no	Yes	Yes	No	Yes	0.58	0.56	0.99	0.61	0.74

**Figure 18:**
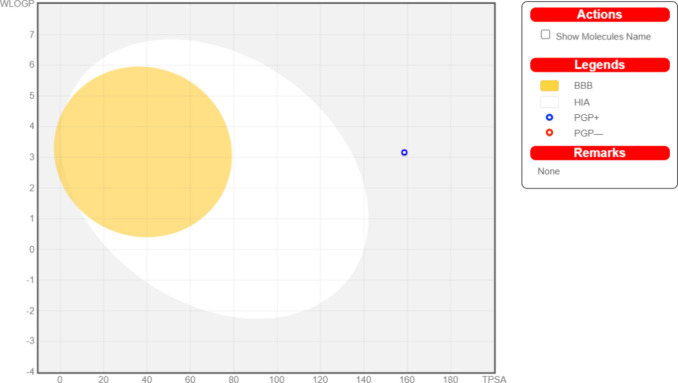
BOILED-Egg plot for **5**.

The plot in Figure 18, resembling a “boiled egg”, illustrates the comparison between the total polar surface area (TPSA) and water–lipid partition coefficient (WLOP). The white and yellow regions on the plot symbolize the passage of compound **5** through the gastrointestinal tract (GIA) and the blood–brain barrier (BBB), respectively. Notably, **5** is identified within the PGP^+^ region based on this plot. A compound falling into this region suggests that it may be a substrate for P-gP, potentially influencing its transport characteristics and distribution in the body. This information is valuable in understanding compound pharmacokinetic behavior.

## 4 Conclusions

To sum up, a new pyrazolo[3,4-*g*]isoquinoline derivative was synthesized, and its structure was confirmed by single-crystal X-ray analysis. Although Hirshfeld surfaces show that intermolecular hydrogen bonds are more important for crystal packing, QTAIM, NCI, and DFT-NBO calculations indicate that intramolecular hydrogen bonds are also present. However, the QTAIM results differ from those of NCI and NBO calculations. QTAIM shows that there is no intramolecular hydrogen bond between N13 and C17. However, the results of NCI and NBO show that there should be a C17–H···N13 intramolecular hydrogen bond. Molecular docking studies show that **5** exhibits a smaller binding affinity on a target protein compared with that of the native ligand. The molecular dynamics result suggests that the **5** and LRRK2 complex system was stable up to 35 ns, and after that, considerable structural changes occurred from 35 to 94 ns, which may be due to loop movement or side chain rearrangement, and finally, after 94 ns, the complex system slowly stabilized with deviations of only about 2 nm. ADMET analysis also reveals that **5** is potentially influencing the *P*-glycoprotein substrate, and other parameters suggest information useful for developing future drug candidacy.

## Supporting Information Available

FTIR, ^1^H-NMR, and ^13^C-NMRd spectrum of compound **5** (Figures S1–S3); crystal and refinement details for **5** (Table S1); Hirshfeld surface property information for **5** (Table S2); fingerprint (%) of the total surface area (Table S3); interactions of LRRK2–ligand complexes (Table S4); and electronic transitions from S_0_ to S_*n*_ (Table S5) (PDF)
