# Supplementary material for: Synthesis, Crystal Structure, Hirshfeld Surface Analysis, and Computational Approach of a New Pyrazolo[3,4-g]isoquinoline Derivative as Potent against Leucine-Rich Repeat Kinase 2 (LRRK2)
Source: ACS Omega. 2024 Jul 1;9(28):30751–70. doi: 10.1021/acsomega.4c03208 (PMC11256088; doi:10.1021/acsomega.4c03208)

Supporting Information

Synthesis, Crystal structure, Hirshfeld surface analysis and computational approach of new pyrazolo[3,4-*g*]isoquinoline derivative as potent against Leucine-rich repeat kinase 2 (LRRK2)

Etify A. Bakhite [1], Shaaban Kamel Mohamed [2,3], Chin-Hung Lai [4,5,*], Karthikeyan Subramani [6], Islam S. Marae [1], Suzan Abuelhassan [1], Abdelhamid A. E. Soliman [1], Mohamed S. K. Youssef [1], Hatem A. Abuelizz [8], Joel T. Mague[7], Rashad Al-Salahi [8], Youness El Bakri [9,*]

[1]Department of Chemistry, Faculty of Science, Assiut University, Assiut 71516, Egypt.
[2] Chemistry and Environmental Division, Manchester Metropolitan University, Manchester, M1 5GD, England.
[3] Chemistry Department, Faculty of Science, Minia University, 61519 El-Minia, Egypt.
[4] Department of Medical Applied Chemistry, Chung Shan Medical University, Taichung 40241, Taiwan.
[5] Department of Medical Education, Chung Shan Medical University Hospital, Taichung 40201, Taiwan.
[6] Center for Healthcare Advancement, Innovation and Research, Vellore Institute of Technology University, Chennai Campus, Chennai, 600127, India.
[7] Department of Chemistry, Tulane University, New Orleans, LA 70118, USA.
[8] Department of Pharmaceutical Chemistry, College of Pharmacy, King Saud University, Riyadh 11451, Saudi Arabia.
[9] Department of Theoretical and Applied Chemistry, South Ural State University, Lenin prospect 76, Chelyabinsk, 454080, Russian Federation.

Corresponding authors: chlai125@csmu.edu.tw; yns.elbakri@gmail.com

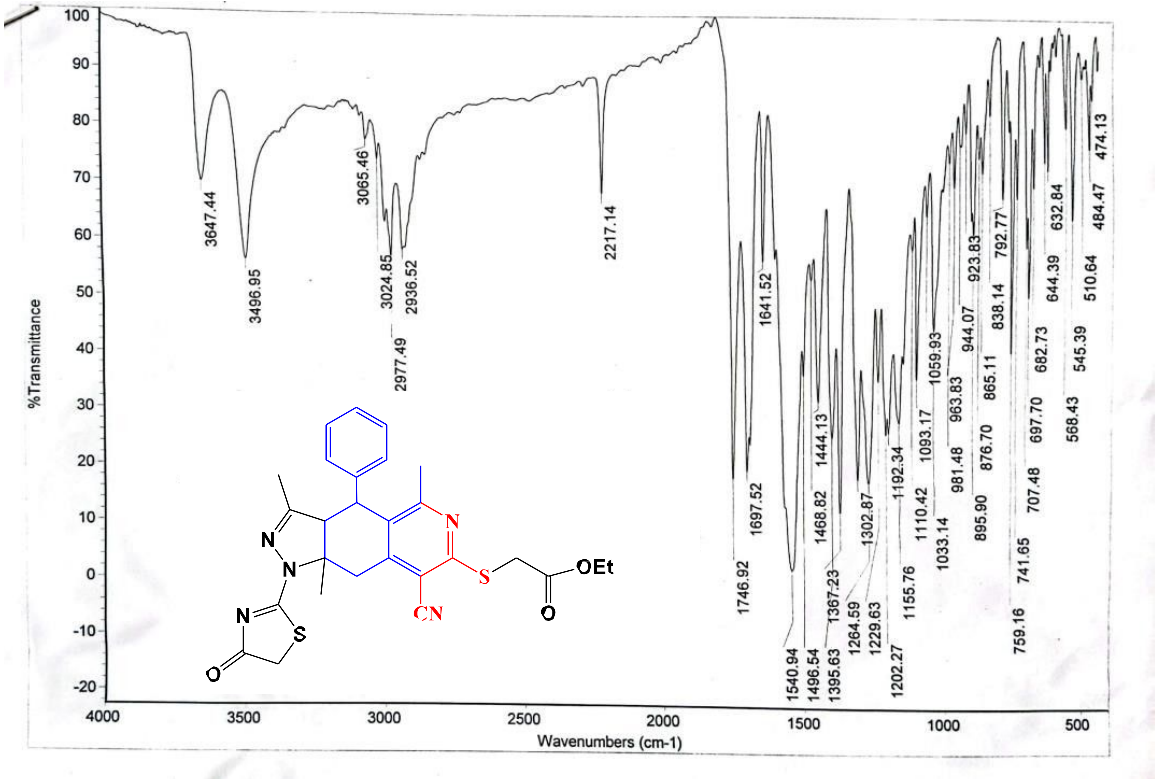

Figure S1. FT-IR spectrum of compound **5**

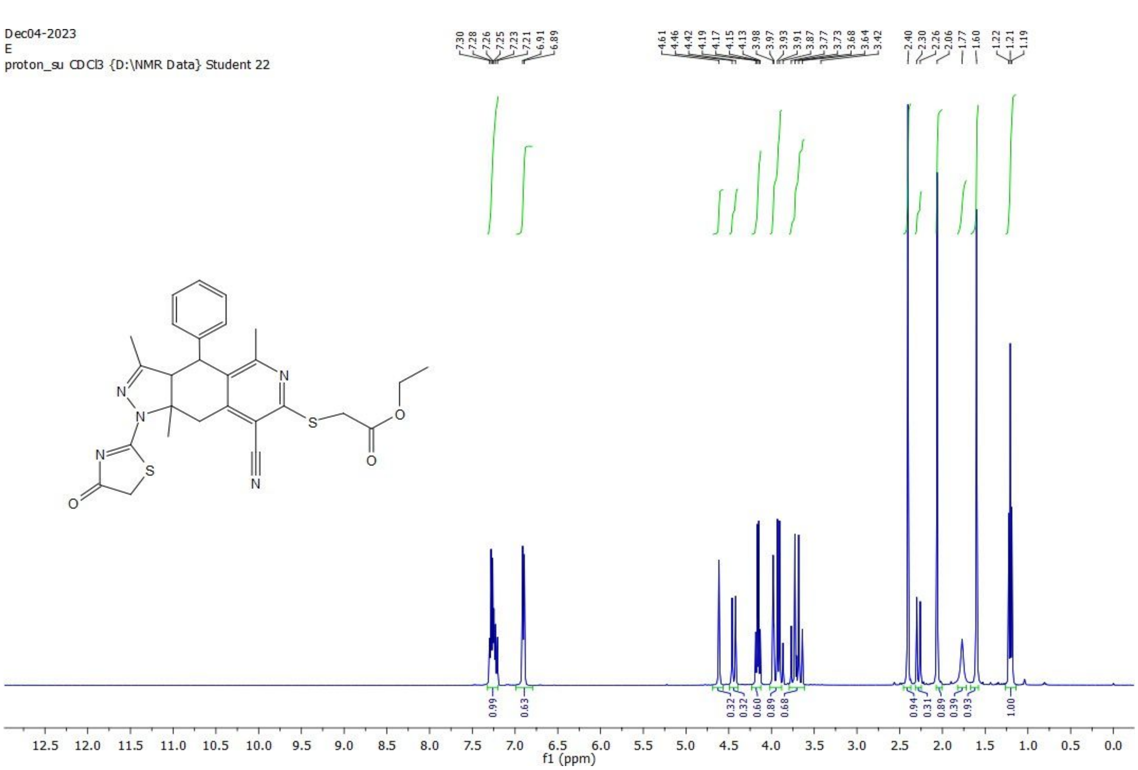

Figure S2. ^{1}H-NMR spectrum of compound **5.**

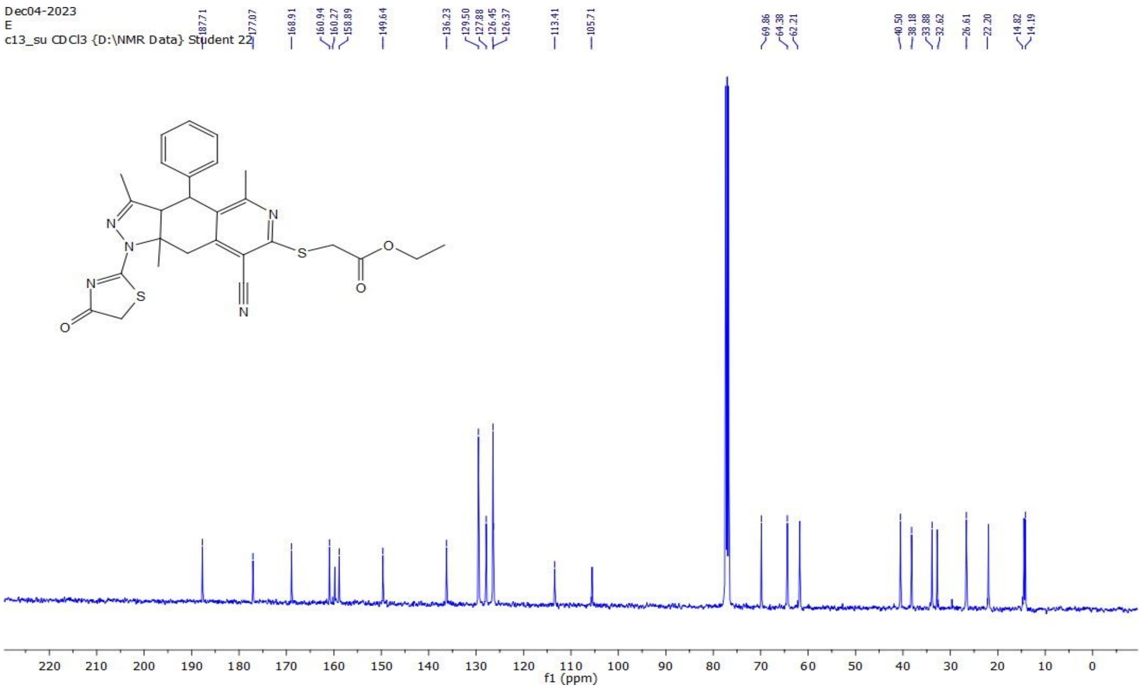

Figure S3. ^{13}C-NMR spectrum of compound **5**

Table S1. Crystal and refinement details for **5**.

Chemical formula	$C_{27}H_{27}N_5O_3S_2 \cdot 0.75(H_2O)$
M_r	547.17
Crystal system, space group	Monoclinic, $P2_1/n$
Temperature (K)	150
a, *b*, *c* (Å)	13.0561(10), 12.6792(9), 16.2488(12)
β (°)	91.628(4)
V (Å^3)	2688.8(3)
Z	4
Radiation type	Mo $K\alpha$
μ (mm^{-1})	0.24
Crystal size (mm)	0.42 × 0.24 × 0.10
Data collection	
Diffractometer	Bruker D8 QUEST PHOTON 3 diffractometer
Absorption correction	Numerical *SADABS*
T_{min}, T_{max}	0.93, 0.98
No. of measured, independent and observed [$I > 2\sigma(I)$] reflections	129732, 7582, 6773
R_{int}	0.031
$(\sin \theta/\lambda)_{max}$ (Å^{-1})	0.696
Refinement	
$R[F^2 > 2\sigma(F^2)]$, $wR(F^2)$, S	0.045, 0.115, 1.09
No. of reflections	7582
No. of parameters	359
No. of restraints	2
H-atom treatment	H-atom parameters constrained
$\Delta\rho_{max}$, $\Delta\rho_{min}$ (e Å^{-3})	0.59, −0.40

Table S2. Hirshfeld Surface property information for **5** (unit: Å).

**Surface**	**Minimum**	**Maximum**	**Mean**
d_{norm}	-0.5362	1.4441	0.4524
shape index	-0.9968	0.9992	0.1816
curvedness	-4.1535	0.5093	-0.9390
fragment patch	0.0000	26.0000	9.0030

Table S3. Fingerprint (%) of the total surface area for closed contact between atoms inside and outside the surface for **5**.

**5**					
Atoms	H	C	N	S	O
H	44.4	11.1	14.8	9.7	13.9
C	11.1	0.9	0.0	2.8	0.5
N	14.8	0.0	0.6	0.0	0.8
S	9.7	2.8	0.0	0.0	0.0
O	13.9	0.5	0.8	0.0	0.6

Table S4. Interactions of LRRK2-ligand complexes.

Protein-Drug complex	Interaction between donor and acceptor	Distance (Å)	Nature of interaction
LRRK2 -5	A:ARG 1483:HH22- d:UNK1:O35	1.68	Conventional hydrogen bond
	A:ARG 1483:HE- d:UNK1:O32	2.16	
	d:UNK1:C21- A:LEU1474	3.68	Alkyl
	A:ALA1481- d:UNK1:C21	3.73	
	d:UNK1:C33- A:ARG1483	4.47	
	d:UNK1:C33- A:PRO1446	4.78	
	A:ALA1481:CB- d:UNK1	3.90	Pi-Sigma
	d: UNK1- A:ARG1483	5.02	Pi-Alkyl
	A:SER1444:O- d:UNK1:O35	2.90	Acceptor-Acceptor
LRRK2 – native ligand	d:UNL1:N-A:GLN1411:O	3.06	Conventional hydrogen bond
	A:LEU1517:HN- d:UNL1:O	2.51	
	d:UNL1:C-A:ARG1412:O	3.10	Carbon hydrogen bond
	d:UNL1- A:LEU1388	5.26	Pi-Alkyl
	d:UNL1- A:VAL1519	4.01	
	d:UNL1- A:MET1335	4.60	
	d:UNL1- A:LEU1388	5.26	
	A:MET1335- d:UNL1:C	3.98	Alkyl
	A:LEU1414- d:UNL1:C	3.66	
	A:ILE1513- d: UNL1	4.43	
	d:UNL1:C- d:UNL1	3.66	Pi-sigma

Table S5. The electronic transitions from S_0 to S_n (n= 1 ~ 20).

n	Excitation wavelength (λ in nm)	Oscillator strength (f)	Contribution	1 → 2	2 → 1
1	315.2	0.0398	HOMO→LUMO (94%) HOMO→LUMO+2 (2%)	0.0807	0.0461
2	308.6	0.0011	HOMO-1→LUMO (94%) HOMO-2→LUMO (5%)	0.0023	0.8940
3	288.3	0.0007	HOMO-2→LUMO (94%) HOMO-1→LUMO (5%)	0.0007	0.9150
4	284.4	0.0005	HOMO-3→LUMO+1 (64%), HOMO-3→LUMO+3 (14%) HOMO-3→LUMO+6 (2%), HOMO-3→LUMO+8 (7%), HOMO-2→LUMO+1 (6%)	0.0012	0.0120
5	277.8	0.0001	HOMO-3→LUMO (99%)	0.0003	0.9139
6	271.2	0.0026	HOMO-1→LUMO+2 (87%) HOMO-2→LUMO+2 (6%), HOMO-1→LUMO+1 (4%)	0.0041	0.8327
7	270.1	0.0345	HOMO → LUMO+1 (94%) HOMO-2→LUMO+1 (2%), HOMO→LUMO+2 (2%)	0.8422	0.0068
8	268.5	0.1669	HOMO-2→LUMO+1 (42%), HOMO→LUMO+2 (42%) HOMO-3→LUMO+1 (3%), HOMO-2→LUMO+3 (2%), HOMO→LUMO+1 (5%)	0.3097	0.1897
9	267.7	0.1456	HOMO-2→LUMO+1 (32%), HOMO-1→LUMO+1 (30%), HOMO→LUMO+2 (25%) HOMO-3→LUMO+1 (4%), HOMO-2→LUMO+3 (2%)	0.2059	0.1835
10	263.9	0.0089	HOMO-7→LUMO (80%), HOMO-4→LUMO (12%) HOMO→LUMO+2 (2%)	0.0695	0.1229
11	263.6	0.3574	HOMO-2→LUMO+1 (12%), HOMO-1→LUMO+1 (59%), HOMO→LUMO+2 (19%) HOMO-1→LUMO+2 (2%)	0.1781	0.1789
12	254.9	0.0033	HOMO-2→LUMO+2 (91%)	0.0014	0.8483

			HOMO-1→LUMO+2 (7%)		
13	253.0	0.0001	HOMO-7→LUMO (12%), HOMO-4→LUMO (84%)	0.0073	0.8366
14	249.2	0.0015	HOMO→LUMO+3 (97%)	0.8871	0.0033
15	246.1	0.0005	HOMO-3→LUMO+2 (96%)	0.0009	0.8458
16	245.8	0.0996	HOMO-1→LUMO+3 (87%) HOMO-2→LUMO+3 (3%), HOMO→LUMO+4 (2%)	0.0430	0.0529
17	244.8	0.0044	HOMO→LUMO+4 (92%) HOMO-5→LUMO (3%), HOMO-1→LUMO+3 (2%)	0.8282	0.0069
18	243.5	0.0014	HOMO-5→LUMO (92%) HOMO→LUMO+4 (3%)	0.0059	0.8256
19	241.1	0.0004	HOMO-1→LUMO+4 (88%) HOMO-5→LUMO (2%), HOMO-2→LUMO+4 (5%)	0.0282	0.0468
20	238.8	0.0033	HOMO-7→LUMO+2 (74%) HOMO-6→LUMO (2%), HOMO-4→LUMO+2 (9%), HOMO→LUMO+5 (2%)	0.1369	0.1338

checkCIF/PLATON report

Structure factors have been supplied for datablock(s) SKM301_0m_a

THIS REPORT IS FOR GUIDANCE ONLY. IF USED AS PART OF A REVIEW PROCEDURE FOR PUBLICATION, IT SHOULD NOT REPLACE THE EXPERTISE OF AN EXPERIENCED CRYSTALLOGRAPHIC REFEREE.

No syntax errors found. CIF dictionary Interpreting this report

Datablock: SKM301_0m_a

Bond precision:	C-C = 0.0019 A		Wavelength=0.71073
Cell:	a=13.0561(10)	b=12.6792(9)	c=16.2488(12)
	alpha=90	beta=91.628(4)	gamma=90
Temperature:	150 K		

	Calculated	Reported
Volume Space	2688.8(3)	2688.8(3)
group Hall	P 21/n	P 21/n
group	-P 2yn	-P 2yn
Moiety formula	4(C27 H27 N5 O3 S2), 3(H2 O)	C27 H27 N5 O3 S2, 0.75(H2 O)
Sum formula	C108 H114 N20 O15 S8	C27 H28.50 N5 O3.75 S2
Mr	2188.67	547.17
Dx,g cm-3	1.352	1.352
Z	1	4
Mu (mm-1)	0.240	0.240
F000	1150.0	1150.0
F000’	1151.42	
h,k,lmax	18,17,22	18,17,22
Nref	7598	7582
Tmin,Tmax	0.933,0.976	0.930,0.980
Tmin’	0.905	

Correction method= # Reported T Limits: Tmin=0.930 Tmax=0.980 AbsCorr = NUMERICAL

Data completeness= 0.998 Theta(max)= 29.647

R(reflections)= 0.0452(6773) wR2(reflections)= 0.1145(7582)

S = 1.094 Npar= 359

The following ALERTS were generated. Each ALERT has the format
test-name_ALERT_alert-type_alert-level.
Click on the hyperlinks for more details of the test.

Alert level B

PLAT420_ALERT_2_B D-H Bond Without Acceptor O4 --H4C . Please Check

---

Alert level C

PLAT042_ALERT_1_C Calc. and Reported MoietyFormula Strings Differ Please Check
Calc: 4(C27 H27 N5 O3 S2), 3(H2 O)
Rep.: C27 H27 N5 O3 S2, 0.75(H2 O)
PLAT220_ALERT_2_C NonSolvent Resd 1 C Ueq(max)/Ueq(min) Range 4.5 Ratio
PLAT222_ALERT_3_C NonSolvent Resd 1 H Uiso(max)/Uiso(min Range . 5.7 Ratio
PLAT230_ALERT_2_C Hirshfeld Test Diff for O3 --C25 ...... 6.4 s.u.
PLAT906_ALERT_3_C Large K Value in the Analysis of Variance 0.600 3.373 Check
PLAT911_ALERT_3_C Missing FCF Refl Between Thmin & STh/L= . 4 Report
2 0 0, 1 1 1, 1 0 3, 2 1 3,
PLAT975_ALERT_2_C Check Calcd Resid. Dens. 0.87Ang From O4 0.41 eA-3

Alert level G

PLAT002_ALERT_2_G Number of Distance or Angle Restraints on AtSite 5 Note
PLAT007_ALERT_5_G Number of Unrefined Donor-H Atoms .... 4 Report
H4C H4D H4E H4F
PLAT045_ALERT_1_G Calculated and Reported Z Differ by a Factor ... 0.250 Check
PLAT066_ALERT_1_G Predicted and Reported Tmin&Tmax Range Identical ? Check
PLAT171_ALERT_4_G The CIF-Embedded .res File Contains EADP Records 3 Report
PLAT175_ALERT_4_G The CIF-Embedded .res File Contains SAME Records 1 Report
PLAT176_ALERT_4_G The CIF-Embedded .res File Contains SADI Records 1 Report
PLAT189_ALERT_3_G A Non-default SAME Restraint Value for First Par 0.0030 Report
PLAT191_ALERT_3_G A Non-default SADI Restraint Value has been used 0.0020 Report
PLAT230_ALERT_2_G Hirshfeld Test Diff for C26 --C27 . 5.2 s.u.
PLAT301_ALERT_3_G Main Residue Disorder ....(Resd 1) 5% Note
PLAT302_ALERT_4_G Anion/Solvent/Minor-Residue Disorder (Resd 2) 100% Note
PLAT302_ALERT_4_G Anion/Solvent/Minor-Residue Disorder (Resd 3) 100% Note
PLAT304_ALERT_4_G Non-Integer Number of Atoms in ..... (Resd 2) 1.93 Check
PLAT304_ALERT_4_G Non-Integer Number of Atoms in ..... (Resd 3) 0.32 Check
PLAT415_ALERT_2_G Short Inter D-H..H-X H6 ..H4E . 1.89 Ang.
-1/2+x,3/2-y,-1/2+z = 4_575 Check
PLAT415_ALERT_2_G Short Inter D-H..H-X H18 ..H4E . 1.83 Ang.

-1/2+x,3/2-y,-1/2+z = 4_575 Check
PLAT480_ALERT_4_G Long H...A H-Bond Reported H4C..S1 . 2.89 Ang.
PLAT790_ALERT_4_G Centre of Gravity not Within Unit Cell: Resd. # 2 Note
H2 O
PLAT790_ALERT_4_G Centre of Gravity not Within Unit Cell: Resd. # 3 Note
H2 O
PLAT860_ALERT_3_G Number of Least-Squares Restraints .... 2 Note
PLAT899_ALERT_4_G SHELXL2018 is Deprecated and Succeeded by SHELXL 2019/3 Note
PLAT910_ALERT_3_G Missing # of FCF Reflection(s) Below Theta(Min). 3 Note
-1 0 1, 0 1 1, 1 0 1,
PLAT912_ALERT_4_G Missing # of FCF Reflections Above STh/L= 0.600 9 Note
PLAT913_ALERT_3_G Missing # of Very Strong Reflections in FCF .... 3 Note
-1 0 1, 1 0 3, 2 1 3,

PLAT933_ALERT_2_G Number of HKL-OMIT Records in Embedded .res File 4 Note
1 0 1, -1 0 1, 0 1 1, 1 1 1,
PLAT969_ALERT_5_G The 'Henn et al.' R-Factor-gap value .... 8.66 Note
Predicted wR2: Based on SigI**2 1.32 or SHELX Weight 10.72
PLAT978_ALERT_2_G Number C-C Bonds with Positive Residual Density. 17 Info

---

0 **ALERT level A** = Most likely a serious problem - resolve or explain
1 **ALERT level B** = A potentially serious problem, consider carefully
7 **ALERT level C** = Check. Ensure it is not caused by an omission or oversight
28 **ALERT level G** = General information/check it is not something unexpected

3 ALERT type 1 CIF construction/syntax error, inconsistent or missing data
10 ALERT type 2 Indicator that the structure model may be wrong or deficient
9 ALERT type 3 Indicator that the structure quality may be low
12 ALERT type 4 Improvement, methodology, query or suggestion
2 ALERT type 5 Informative message, check

It is advisable to attempt to resolve as many as possible of the alerts in all categories. Often the minor alerts point to easily fixed oversights, errors and omissions in your CIF or refinement strategy, so attention to these fine details can be worthwhile. In order to resolve some of the more serious problems it may be necessary to carry out additional measurements or structure refinements. However, the purpose of your study may justify the reported deviations and the more serious of these should normally be commented upon in the discussion or experimental section of a paper or in the "special_details" fields of the CIF. checkCIF was carefully designed to identify outliers and unusual parameters, but every test has its limitations and alerts that are not important in a particular case may appear. Conversely, the absence of alerts does not guarantee there are no aspects of the results needing attention. It is up to the individual to critically assess their own results and, if necessary, seek expert advice.

Publication of your CIF in IUCr journals

A basic structural check has been run on your CIF. These basic checks will be run on all CIFs submitted for publication in IUCr journals (*Acta Crystallographica*, *Journal of Applied Crystallography*, *Journal of Synchrotron Radiation*); however, if you intend to submit to *Acta Crystallographica Section C* or *E* or *IUCrData*, you should make sure that full publication checks are run on the final version of your CIF prior to submission.

Publication of your CIF in other journals

Please refer to the *Notes for Authors* of the relevant journal for any special instructions relating to

CIF submission.

PLATON version of 06/01/2024; check.def file version of 05/01/2024

Datablock SKM301_0m_a - ellipsoid plot

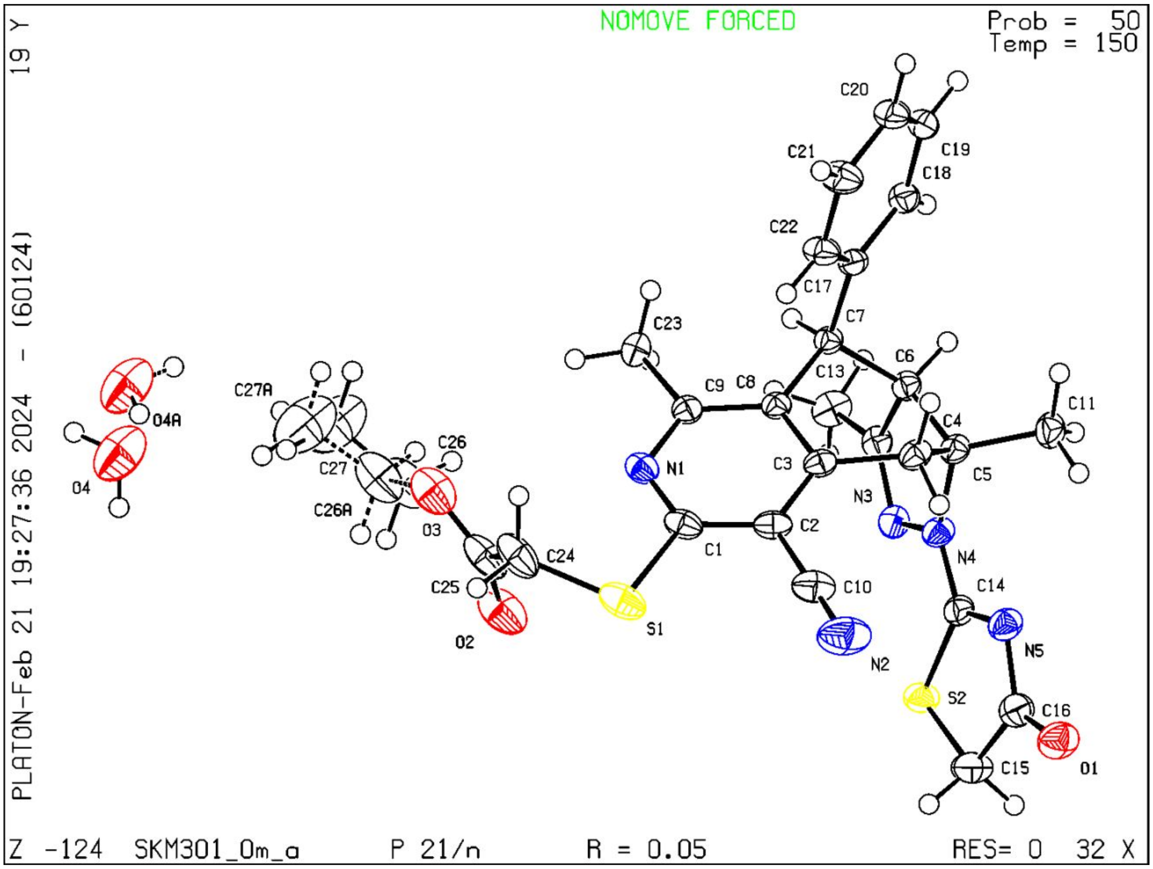

Supplement: Supplementary file 1 — ao4c03208_si_001.pdf [file ao4c03208_si_001.pdf]
